# Household income and women's reproductive strategies in Almaty (Kazakhstan): urban stratification of access to health

**DOI:** 10.1016/j.dialog.2026.100355

**Published:** 2026-09-29

**Authors:** Ainur Bakytzhanova, Gulzhan Alimbekova

**Affiliations:** a Department of Sociology and Social Work, Al-Farabi Kazakh National University, Almaty, Kazakhstan; b Public Opinion Research Center, Almaty, Kazakhstan

**Keywords:** Reproductive determinants, Household material well-being, Geography of opportunities, Infrastructure provision and safety, Territorial inequality, Access to medical care

## Abstract

**Purpose:**

The aim of this article is to demonstrate how household financial status and the spatial structure of opportunities in the metropolis are interrelated with women's reproductive strategies in Almaty, where formal access to healthcare is combined with uneven access costs in terms of time, money, quality, and predictability of service delivery.

**Methods:**

The empirical basis consisted of a cross-sectional survey of women of reproductive age in eight districts of Almaty (*N* = 934). The analysis utilized a scale reliability assessment (Cronbach's α), χ^2^ contingency tables with Cramer's V, and interdistrict comparisons using the nonparametric Kruskal-Wallis test and the median test. The urban environment was operationalized through an infrastructure provision index, comprising six domains: Education, clinics, retail and service environment, leisure, sports, and safety.

**Results:**

The resulting six-item index demonstrated good internal consistency (Cronbach's α = 0.814; standardized α = 0.815; *n* = 780). Statistically significant interdistrict heterogeneity was found in accessibility of clinics (χ^2^ = 16.048; *p* = 0.025), retail and service infrastructure (χ^2^ = 28.472; *p* < 0.001), leisure (χ^2^ = 32.640; *p* < 0.001), sports (χ^2^ = 31.666; *p* < 0.001), and safety (χ^2^ = 17.801; *p* = 0.013), while no differences were found in educational infrastructure (*p* = 0.844). The most significant predictors of reproductive practices and attitudes were household financial well-being and education level. The results demonstrate that the formal availability of services does not imply equality in actual access to them.

**Conclusion:**

Inequality in reproductive opportunities is reproduced through hidden transaction costs of access, waiting, transportation logistics, the need for paid alternatives, and additional support resources. Neighborhood of residence is not only a territorial characteristic but also an indicator of the organizational structure of everyday life, influencing care-seeking behavior and family planning strategies. The proposed framework of the Atlas of Reproductive Opportunities can be used to identify local bottlenecks and inform targeted preventive and management decisions.

## Highlights

• Household income significantly influences access to reproductive care.
• Formal availability of services does not mean equal access in Almaty.
• Housing instability increases barriers to accessing healthcare.
• Residential neighborhood influences women's reproductive options.
• Hidden costs of time and money exacerbate health inequalities.

## Graphical abstract

**Figure:**
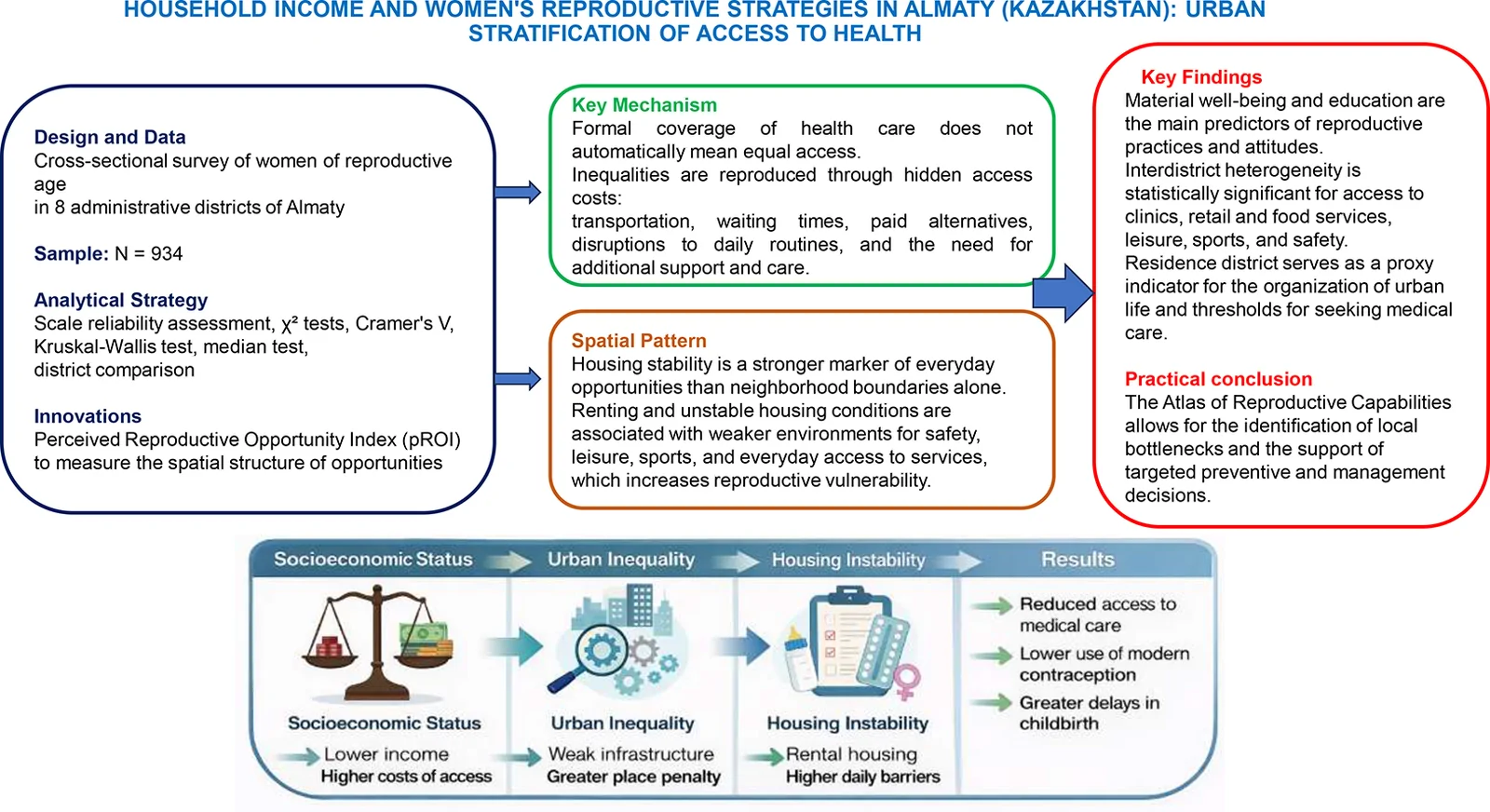

## 1 Introduction

Kazakhstan today represents a paradoxical social model in which sustained economic growth and macroeconomic stability are not accompanied by an equivalent improvement in reproductive and maternal health indicators. Over the past two decades, the government has demonstrated significant success in digitalizing medicine, expanding infrastructure, and modernizing perinatal care, but structural inequalities in access to quality medical services remain a chronic problem.

The analysis of administrative statistics on the indicator in Kazakhstan for 2010-2024 records not just a decrease in maternal mortality, but a steady intra-stratification by territory and type of settlement. At the national level, the maternal mortality rate decreased from 22.7 to 10.1 per 100,000 live births (2010 → 2024), but the trajectory contains a shock gap in 2020-2021: An increase from 13.7 (2019) to 36.5 (2020) and 44.7 (2021), that is, an increase of more than 3 times relative to the pre-crisis level (Fig. 1) [1], [2]. This can be seen from two empirical markers: (1) By 2024, the rural-urban gap had acquired a stable social sign of 13.5 in rural areas versus 9.3 in urban areas (+45% of the urban level); (2) The regional scope in 2024 reaches 8-9 times its size (from 4.2 in Almaty to 36.5 in the region of Zhetisu), which indicates a structural heterogeneity of risks and managerial effectiveness at the subnational level (Fig. 1). Megacities do not exhibit linear “opposite dynamics,” but rather high volatility and sensitivity to system load (for example, Almaty: 15.4 → 53.8 → 4.2 in 2019 → 2021 → 2024), which is consistent with the effects of migration pressure, segregation of access, and concentration of complex clinical cases in large centers.

**Fig. 1:**
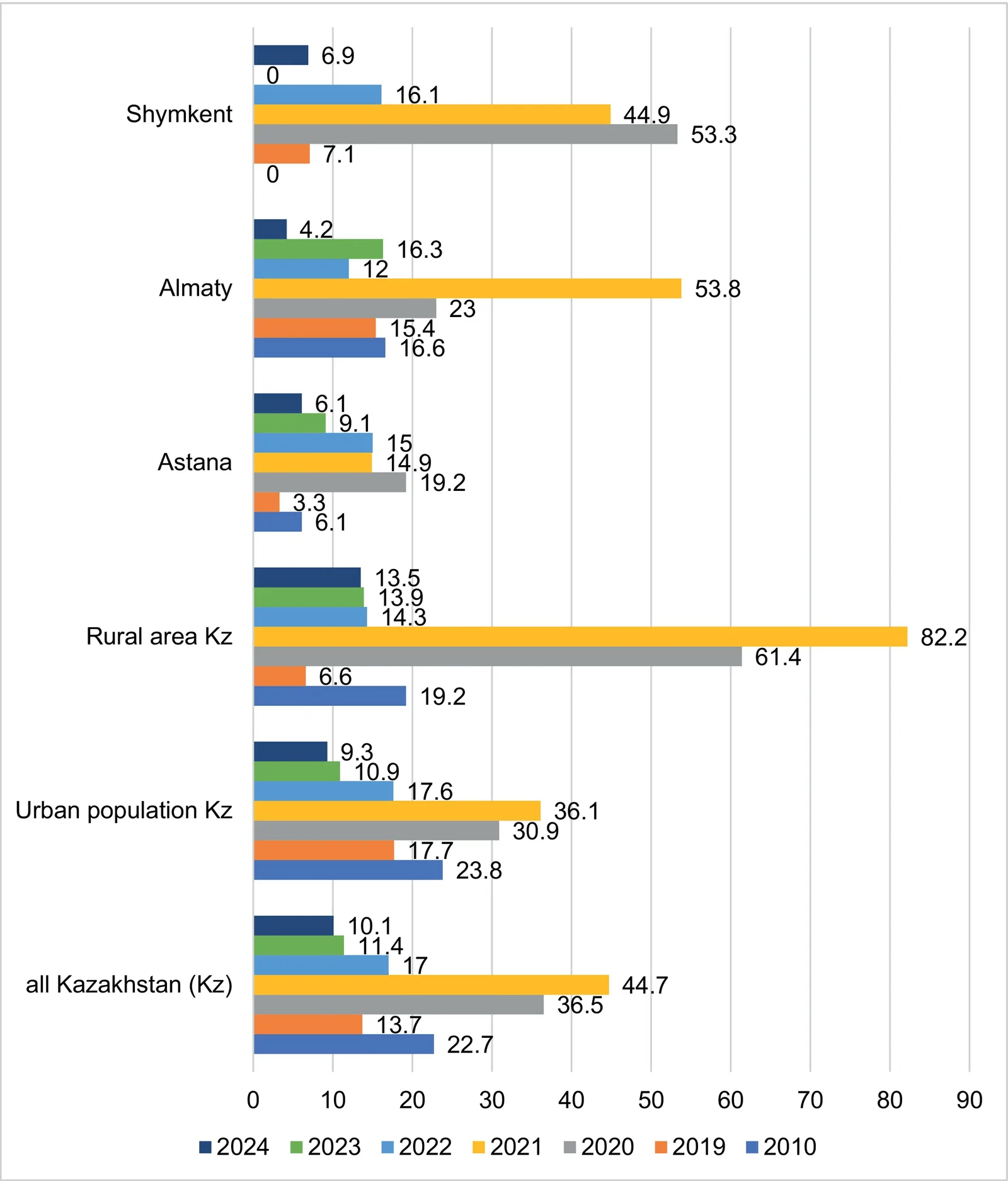
Maternal mortality: Trend, rural-urban gap and territorial scale (CMR per 100,000 live births) [1].

Understanding the nature of these differences requires going beyond the traditional analysis of social determinants and addressing spatial and institutional aspects. In this regard, the methodological integration of the Opportunity Atlas approach proposed by the Raj Chetty research group is of particular importance [3]. This approach makes it possible to analyze the impact of places of residence on a person's life and economic outcomes, revealing how spatial differences form unequal access to resources and opportunities.

Even with the target threshold of SDG 3 (<70 per 100,000 live births by 2030), there remains a sharp disparity in outcomes: In 2020, almost 95% of maternal deaths occurred in low- and lower-middle-income countries, where the rate was 462 per 100,000 versus 11 in high-income countries [4], [5], [6]. Against the background of a general decrease in maternal mortality by 34% in 2000-2020, violations in 92% of health systems demonstrated how much this progress depends on the sustainability of infrastructure and the availability of care [7], [8], [9]. Kazakhstan, as a middle-income country, is faced with the fact that maternal health outcomes are determined not only by the healthcare system, but also by socio-demographic determinants: In national and international studies, they consistently include age, marital status, education level, frequency of prenatal visits and staffing [10].

Against this background, despite the reforms, there remains a gap with OECD countries in a number of key health indicators [11], and a significant proportion of direct household spending continues to limit access to health services for vulnerable groups [12], [13], [6].

### 1.1 Research questions, hypotheses, and contributions to research

Almaty, the largest megacity in Kazakhstan, demonstrates a characteristic gap between formal medical coverage and effective access to medical care. Previous studies in Kazakhstan have documented persistent socio-economic inequality in the field of healthcare and have shown that out-of-pocket expenses can limit access to medical care, despite formally universal coverage [12], [31]. These results indicate that household financial resources can influence not only the ability to receive medical care, but also the ability of women to translate reproductive intentions into real practice. International studies also show that socio-economic resources are linked to access to reproductive health care, the use of contraceptives, and the ability to plan the timing of childbearing. [7], [9], [29], [43]. At the same time, access is linked to space: the place of residence determines access to infrastructure, services, transportation, security, and other resources that may facilitate or limit the use of medical services and reproductive opportunities [14], [15], [16], [17], [18], [34], [35]. Collectively, this literature provides a framework for exploring whether household resources and the inner-city context are separate but interrelated aspects of reproductive inequality in Almaty. Accordingly, the study addresses the following research questions:

***RQ1.*** How is household financial status related to women's reproductive health in Almaty, including the use of modern contraceptive methods, financial denial of medical care, postponing childbearing, and reproductive health awareness?

***RQ2.*** Do the observed associations between household resources and women's reproductive attitudes and practices persist after controlling for age, education, employment, marital status, number of children, district of residence, and length of residence in Almaty?

***RQ3.*** Is the spatial context of residence, including district and perceived infrastructure conditions, associated with differences in access to care and reproductive practices among women in Almaty?

Based on the evidence discussed above, the hypotheses determine the expected direction of the relationship between household financial resources, reproductive practices, and spatial context. Preliminary data on socio-economic inequality in access to health services confirm the assumption of stronger financial barriers among women with fewer household resources [12], [31], [43], while studies on reproductive autonomy and access show that greater socio-economic resources empower women to obtain contraception and implement reproductive plans [7], [9], [29]. Research based on geography and neighborhood also shows that opportunities are structured not only based on individual resources, but also based on the infrastructural and socio-economic characteristics of the environment in which people live. [14], [30], [34], [35]. On this basis, the following hypotheses were tested:

H1 (income and access barriers) Women from households with lower levels of financial well-being are statistically more likely to report financial barriers when accessing reproductive health services.

H2 (income and reproductive practices) Higher levels of household financial well-being are statistically associated with a higher likelihood of using modern contraceptive methods and with more pronounced reproductive planning practices.

H3 (spatial context) The spatial context of residence, reflected through neighborhood and perceived infrastructure conditions, is statistically associated with differences in reproductive opportunities and access to care, even after accounting for key sociodemographic characteristics.

Reproductive behavior in urban Kazakhstan is increasingly manifested not as a purely “private decision,” but as a form of adaptation to risks associated with income, the cost of housing and care, access to healthcare, and the quality of the urban environment. Therefore, the question of reproductive strategies is linked not only to demographic indicators but also to quality of life: It reflects the predictability of life trajectories and the even distribution of opportunities for maintaining health. This article examines how household financial status is associated with access to reproductive care and family planning practices, and also analyzes how the spatial context of residence relates to differences in women's reproductive opportunities. This approach allows us to move beyond simply describing differences between groups to explaining how socioeconomic and spatial conditions are associated with the reproduction of inequalities in reproductive health.

## 2 Conceptual framework and literature review

### 2.1 Reproductive health and spatial inequality

Contemporary literature increasingly views reproductive health as a product of the intersection of bodily processes, social relations, and the spatial organization of everyday life, moving the analysis beyond biomedical reductionism. In this perspective, health is shaped not “within the body” as an autonomous object, but through networks of infrastructure, social support, and localized access to resources. Within the framework of feminist geography, Pettway et al. demonstrate that reproductive experiences are rooted in space – from intimate practices and everyday routes to the power structures that determine who receives medical care, information, and safety, and under what conditions [14].

A related field, the political ecology of health, shifts the focus to material and environmental conditions as active mechanisms for the reproduction of inequalities. Nading argues that the environment/place does not function as a passive background, but as an agent through which vulnerabilities are fixed [15]. Toxic effects are intertwined with institutional decisions, economic constraints, and unequal risk visibility [16]. In the logic of ecological reproductive justice, Hoover, link environmental threats to issues of rights, responsibility, and resource allocation, emphasizing that reproductive well-being should be understood as a socially protected right, and not as an individual's “ability to cope” [17].

At the same time, a number of researchers point to the methodological risk of the ecological framework: With an emphasis on “toxicity”, space can be interpreted too instrumentally as a carrier of influence, while the cultural meanings of the place, bodily subjectivity and symbolic modes of belonging remain in the shadows. Nightingale emphasizes that the relationship between body and place cannot be reduced to physical exposure [18]; rather, this relationship is formed through social practices, knowledge, norms, and local hierarchies that determine how risk is experienced, interpreted, and transformed into behavior [16], [17].

### 2.2 Reproductive geography: from body to territory

The distributive approach in modern literature describes the reproductive environment not as a set of individual conditions, but as a socio-spatial system in which everyday intimate practices, local infrastructure, and political and institutional decisions form an interdependent circuit of reproduction opportunities. Thus, Calkin conceptualize the reproductive environment as a spatial architecture of resources and constraints, where access consists of a combination of services, norms, and management regimes in the territory [19], [20]. In a similar logic, Pain emphasizes that the key analytical object is not a single service, but the spatial mode of everyday life, a set of routes, security boundaries, and institutional entry points through which the real achievability of support is determined [21], [19].

Applied research clarifies the mechanisms through which social marginalization translates into inequality of reproductive opportunities. Rivera-Amarillo and Camargo empirically show that differences between territories are manifested not only in the physical accessibility of services, but also in their hidden cost, time losses, transportation costs, bureaucratic barriers and risks of stigmatization, which asymmetrically affect vulnerable groups [22]. In other words, inequality is formed where seeking help becomes “expensive” not only financially, but also socially through loss of time, status and security.

Even in the context of reforms and the expansion of the formal supply of services, spatial gaps often prove to be persistent. González-Vélez and Jaramillo-Sierra note that institutional changes do not automatically eliminate the asymmetry between the center and the periphery: The quality of support, continuity of services and consistency of assistance routes continue to vary depending on the territory [23]. Similarly, Herrick shows that differences between urban and non-urban contexts are reproduced through unequal infrastructural resources, human resources and managerial density, as a result of which equal rights give unequal real opportunities [24].

At the micro level, a woman's body turns out to be part of the “landscape” of the material and symbolic. The ecological environment, housing, transport and social infrastructure are included in the reproduction process itself. At the macro level, laws, medical standards, and institutional practices shape the geography of access and denial.

This logic is reinforced by the direction of the political ecology of health and ecological reproductive justice. Hoover and Nading show that environmental pollution regimes, toxic exposures, and industrial zones are part of reproductive rights: Risk is distributed socially and geographically, so “vulnerability” has an address. This is important for urban Kazakhstan, where environmental pressures and social heterogeneity often overlap: Some areas receive more pollution and less infrastructure, while others do the opposite [17], [15]. In this perspective, a woman's body becomes a place where the city literally leaves its mark: Chen, Nading and Hoover describe embediment as a process in which environmental influences and social stress turn into biologically significant consequences affecting the reproductive cycle and health [25], [15], [17].

However, one of the main blind spots in research is the clinical bias. Dyck and Herrick point out that medicine often turns into the only “point of truth”, and everything that happens outside the clinic (home, neighborhood, routine care, transportation, food, water, family burden) disappears from the explanation [26], [20]. However, it is the daily geography that determines whether prevention will take place and whether medical treatment will be timely, especially when household resources are limited [27], [15]. In a megalopolis, this is especially harsh: Access is measured not by the availability of a service on a map, but by the ability to actually reach it in terms of time, money, queues, and communication.

The institutional layer is also not neutral. Freeman, Jeffrey, and Calkin et al. show that rules, discourses, and biomedical regimes produce not only treatment, but also subjectivity: Who is easier to get help, who is more likely to be denied, who faces stigma, and who is supported [27], [28], [19]. Therefore, differences in access to contraception, prenatal care, and reproductive services are often not a personal choice, but the effect of institutional settings and urban segregation of services [25], [27], [29].

Further, literature about the ill-being of the neighborhood acts as a bridge between the city and the body. Nour and Turdybekova et al. show in their research that the socio-economic deprivation of a territory (income, employment, housing, education) is associated with chronic stress and allostatic stress, and through them with a deterioration in reproductive outcomes and limited planning opportunities [29], [30]. This justifies the inclusion of territorial indices of disadvantage and stress channels in the analysis: In cities, inequality lives not only in household income, but also in a place where this income is forced to compensate for the lack of infrastructure.

Finally, the macro context sets the framework, but does not eliminate intra-urban gaps. Spankulova et al. and Liu et al. show that financing, infrastructure, and institutions affect maternal mortality and system quality, but the effects may be ambiguous precisely because aggregated improvements do not guarantee equal access within a country [31], [32]. In the case of Kazakhstan, this is especially important against the background of formally universal guarantees: In the presence of GOBMP/CSHI, real access is often limited by hidden barriers to information, communication, personnel and out-of-pocket costs. Mussabekova et al. and Bukatov et al. describe these barriers as structural and systemically reproducible, especially for vulnerable groups, which makes universality in practice heterogeneous [33], [12].

That is why in this article, the logic of Opportunity Atlas is used not as rhetoric, but as an analytical tool: It allows us to show exactly where a megacity turns social inequality into reproductive restrictions through access/refusal of care, through differences in contraceptive practices and through the cost of pregnancy planning in different urban environments.

#### 2.2.1 The conceptual framework of spatial possibilities

Modern research on inequality is increasingly based on spatial optics, showing that education, income, and health are shaped not only by individual resources, but also by the geography of chances. For example, Chetty et al. demonstrate that the place of childhood and residence is systematically linked to mobility trajectories, and Hendren and Katz show that the effects of public policies and institutions are unevenly distributed across territories [34], [35]. In this logic, Opportunity Atlas suggests measuring place-based differences through: The spatial level (concentration of opportunities/barriers and access to infrastructure); the socio-economic level (family income, parental employment, education, gender, ethnicity as mechanisms of intergenerational mobility); the level of observed life outcomes, which captures how the environment is converted into different trajectories under similar macro conditions.

The Sen and Nussbaum opportunities approach sets a normative framework: Reproductive health is interpreted as a woman's ability to implement reproductive plans with agency and institutional support. At the same time, Opportunity Atlas does not act as a theory of reproductive behavior, but as an empirical place-based logic that allows operationalizing the influence of a place through a comparison of territorial conditions, resource availability, and differences in reproductive practices.

Bronfenbrenner's ecological systems concept is a complementary, multi-level structure that allows us to consider decisions related to reproductive health in the context of interacting social environments. At the microsystem level, reproductive practices are linked to family relationships, dynamics within the relationship with a partner, and direct social support; at the *meso*- and exosystem levels, they are shaped by the influence of medical institutions, service sector infrastructure, working conditions, and other organizational factors; and the macrosystem includes broader gender norms, religious and cultural values, as well as government policy in the field of reproductive health [36], [37], [38]. The ecological concept does not view these levels as independent determinants but emphasizes their interaction: the practical value of individual and family resources partly depends on the institutional and regulatory environment in which they can be used. In this study, this concept complements the approach based on the concept of empowerment and the spatial approach, examining women's reproductive capabilities in the context of social, institutional, and spatial factors.

Epstein shows that biomedicine has long focused on reproductive organs as an explanation of the female body, while systematically excluding women from clinical trials and making the male body an unspoken standard of evidence [39]. In response to this asymmetry, institutional reforms in the United States, the creation of the ORWH, and the NIH Revitalization Act (1993) established the requirement to include women and minorities in research.; However, as Mirin notes, even with progress in oncology, cardiology, and obstetrics, attention and funding remain skewed in favor of “non-feminine” agendas and male selections [40]. In a later framework, Clayton and Collins argue for the transition from formal inclusion to structural solutions: Taking gender into account as a biological variable already at the preclinical level and intersectional analysis in order to eliminate not only the gap of participation, but also the “gap of explanation” of differences in outcomes [41].

### 2.3 Women's health status and spatial determinants of reproductive inequality

Women's reproductive health reflects not only medical, but also socio-spatial differences that form stable trajectories of inequality. Even with high levels of government spending and institutional attention, systemic gaps in gender, income, and place of residence persist [42]. The global goal of SDG-3 is to reduce the maternal mortality rate to below 70 per 100,000 live births by 2030. This remains unattainable for most low- and middle-income countries. According to WHO data, about 95% of all maternal deaths occur in these countries, which indicates a stable relationship between socio-economic status and the likelihood of safe motherhood [7], [9].

However, statistics do not explain the mechanisms of how exactly structural factors, infrastructure, and the microenvironment are converted into risk or protection. That is why in recent years the focus has shifted from the analysis of “individual determinants” to the study of spatial conditions of reproductive health, where not only medical, but also social, environmental and institutional characteristics of the place play a key role.

#### 2.3.1 Socio-spatial environment and reproductive outcomes

The accumulated body of research shows that the unfavorable neighborhood environment, limited employment opportunities, low level of education, poor quality of housing and infrastructure creates chronic stress and allostatic load, which increase the likelihood of pregnancy complications and reduce fertility. These effects are enhanced by limited mobility and the inability to move to “areas of opportunity.” In this regard, the Area Deprivation Index (ADI) has acquired special importance, it is a standardized indicator of territorial deprivation, reflecting the totality of socio-economic and infrastructural deficits [6].

#### 2.3.2 Financing, urbanization and spatial justice in Kazakhstan

In low- and middle-income countries, government spending on healthcare is an important predictor of maternal mortality dynamics, although research results are mixed. Theoretically, this fits into the logic of human capital and the demand for health [10]: Investments in infrastructure, prevention and human resources reduce mortality and strengthen the overall human potential. However, financial injections do not guarantee equality of chances if they are not accompanied by spatial justice, taking into account the urban heterogeneity of access to services and the quality of the environment.

#### 2.3.3 From social determinants to the geography of opportunity

The modern theoretical shift consists in the transition from an abstract understanding of the “social determinants of health” to a more operational concept of the geography of opportunities (Opportunity Atlas) [3], [34], [35]. This framework considers space not as a background, but as an active resource in which the trajectories of human development are formed [43], [44].

The transfer of this approach to the context of urban Kazakhstan, using the example of Almaty, the country's largest megacity, opens up a new level of analysis of reproductive determinants. The Reproductive Opportunity Index can combine the parameters of access to medical care, infrastructure, transport, security, digital connectivity, and social support, turning the normative concept of equity into a measurable spatial indicator.

Thus, unlike the traditional focus on medical indicators, this approach allows us to consider women's health as a result of the interaction of macroeconomic investments and micro-level conditions of everyday life. This creates the basis for targeted policies, from spatial investment targeting to targeted programs to improve health literacy and trust in the healthcare system.

#### 2.3.4 The sociocultural context of reproductive health in Kazakhstan

Kazakhstan represents a specific sociocultural context for the study of reproductive health, as reproductive decisions are shaped by the intersection of post-Soviet modernization, the revival of religiosity, stable family norms, and transforming gender roles. Despite the constitutionally закреплённый secular nature of the state, religious affiliation and culturally rooted family traditions retain social significance. According to the data from the 2021 National Population Census of the Republic of Kazakhstan, out of 19.19 million residents of the country, 13.30 million (69.3%) indicated Islam as their religious affiliation, and 3.30 million (17.2%) indicated Christianity. At the same time, the vast majority of those who indicated Christianity identified themselves as Orthodox 3.27 million people, or 17.0% of the country's total population [45]. These data indicate a pronounced religious heterogeneity in Kazakhstani society, which must be taken into account when considering the sociocultural environment of reproductive decisions. The modern religious landscape of the country has been shaped by the influence of Soviet policy aimed at restricting organized religious life and the subsequent religious revival after gaining independence. In particular, the post-Soviet period was accompanied by an increase in religiosity among ethnic Kazakhs and a growing significance of Islam as both a religious and an ethnocultural marker [46], [47], [48]. At the same time, religious self-identification should not be equated with actual religious practice: studies of the urban population of Kazakhstan show significant differences in the degree of religiosity both within the Muslim and Orthodox-Christian populations and between these groups [49], [50].

These religious and cultural transformations are closely intertwined with the organization of family life and reproductive behavior. Studies conducted in Kazakhstan have documented persistent differences between the country's main ethno-religious groups in terms of reproductive intentions, transitions to having subsequent children, contraceptive practices, and timing of childbearing [51], [52], [53], [54]. Ethnic Kazakh families historically have relatively strong kinship networks and family-related ties, which means that reproductive decisions may potentially depend not only on individual preferences but also on expectations formed within the broader framework of family relationships [55]. This is particularly important because decisions related to birth control depend simultaneously on material capabilities and the normative acceptability of the corresponding behavior. Kan (2024), using the conceptual model Ready–Willing–Able (“readiness–desire–ability”), shows that the relationship between religious affiliation and contraceptive behavior in Kazakhstan must be examined through several interrelated mechanisms, including reproductive intentions, the degree of religiosity, family values, education, employment, and material well-being [55]. Thus, religion is not so much an isolated determinant as part of a broader configuration of norms and socio-economic conditions that shape reproductive preferences and practices.

National data also indicate that family and reproductive decisions in Kazakhstan continue to be shaped by a pronounced normative influence. In the National Report “Kazakhstani Families - 2020,” family, health, material well-being, faith, and religion are listed among the values noted by young people, while respect for elders and spiritual development are also among the values transmitted within the family [56]. The report also reflects the ongoing changes in gender and family roles: women are increasingly combining employment, career aspirations, family responsibilities, and other social roles, while expectations regarding men remain largely tied to the role of the primary breadwinner. Of particular importance is the experts' conclusion that decisions regarding the number of children depend on a combination of interrelated factors, including religion, education, place of residence, age, and level of material well-being [56]. These observations suggest that reproductive behavior cannot be reduced solely to either cultural preferences or economic resources; it is shaped at the intersection of both. The persistent gender organization of family life is also reflected in the results of national studies, according to which most respondents assign the mother a leading role in raising children [56].

An important feature of Kazakhstan's reproductive culture remains the post-Soviet transformation of methods of birth control. In the 1990s, the country was transitioning from a model in which abortion played a significant role in birth control to a more widespread use of contraception. Westoff describes this process as a gradual replacement of abortion with contraceptive practices, whereas Agadjanian et al. show that during the transitional period, both strategies continued to coexist [57], [58]. Between 1995 and 1999, the prevalence of contraception among married Russian women increased from 65% to 70%, and among married Kazakh women from 54% to 64%; at the same time, differences in the use of abortion persisted [55]. Consequently, modern reproductive behavior in Kazakhstan was not shaped from scratch, but rather on the basis of institutionally and culturally entrenched practices of the Soviet system of birth control. A separate dimension of inequality is access to reproductive information. A study by Buckley, Barrett, and Adkins (2008), focusing on young women in Kazakhstan, revealed differences in access to information about reproductive health depending on the channels through which it is obtained; belonging to a non-Kazakh ethnic group was associated with a greater likelihood of having access to information about family planning [50]. This result is fundamentally important because it shows that inequality arises even before a woman directly contacts a doctor: the information resources through which a woman learns about contraception, prevention, and family planning options may differ. Thus, reproductive autonomy depends not only on the availability of medical services but also on the ability to obtain reliable knowledge necessary for making an informed choice and navigating the assistance system [59].

The persistent discrepancy between reproductive intentions and actual outcomes is also evident at the level of unplanned pregnancies. According to estimates by the Guttmacher Institute, in 2015–2019, approximately 696,000 pregnancies occurred annually in Kazakhstan, of which about 259,000 were unplanned, and 212,000 ended in abortion; UNFPA, in turn, estimated the contraceptive prevalence rate among women aged 15–49 at 43% [60], [61]. These indicators point to a deeper problem than the mere formal availability of birth control methods: there remains a gap between the intention to avoid pregnancy and the ability to consistently realize that intention.

For this study, this context is of fundamental analytical importance, since socio-economic resources do not operate independently of the normative environment in which women make reproductive decisions. Household income can determine the financial ability to seek counseling, undergo diagnostics, use contraception, or receive treatment; however, the perceived need to seek medical care, the willingness to do so, the timing of childbearing, the choice of contraceptive methods, and the prioritization of spending on reproductive health may simultaneously be shaped by family expectations, gender roles, religious orientations, and culturally rooted ideas about motherhood and fertility. Consequently, in this study, inequality in the field of reproductive health in urban Kazakhstan is conceptualized as the result of the interaction of material resources, institutional opportunities, and sociocultural conditions, rather than as a consequence of the household income level alone.

From the perspective of ecological systems theory, these factors operate at interconnected levels rather than as separate determinants: household resources and family relationships constitute a woman's immediate reproductive environment, while medical institutions, the urban environment, cultural norms, religion, and government policies form a broader context within which reproductive choice becomes more or less possible [36], [37]. This multi-level approach is particularly relevant for Almaty, where women living under the same state policy may face different household, institutional, and spatial conditions that affect their reproductive opportunities.

#### 2.3.5 National policy in the field of reproductive health and unequal realization of reproductive rights in Kazakhstan

Kazakhstan occupies a special place in the regional system of reproductive health protection. The state is gradually integrating issues of family planning, maternal health protection, infertility treatment, and reproductive technologies into the healthcare system. This focus aligns with Kazakhstan's obligations under the CEDAW and the 1994 International Conference on Population and Development. Kazakhstan ratified the CEDAW in 1998, officially recognizing women's right to freely and responsibly make decisions about the number of children and the intervals between their births, as well as to have equal access to family planning services. The country also assumed obligations under the Cairo Programme of Action and subsequent goals concerning universal access to sexual and reproductive health [62]. Thus, reproductive health is institutionally recognized as a right to medical care, rather than as solely a private or family matter.

Reproductive health policy in Kazakhstan covers contraception, prevention and treatment of infertility, ART, and legal early-term abortion [62; 63; 64]. High coverage of maternal care: 99.3% of women received qualified prenatal care, and 99.4% of births took place with the participation of qualified specialists [65]. Since 2023, preconception care has been developing; in Almaty, in 2025, nearly 673 million tenge was allocated for it through the Social Health Insurance Fund [66]. The “Analar saulygy” program also provides for preparation for pregnancy and early detection of diseases [65]. However, formal availability of services does not mean their actual availability. UNFPA identifies women with low incomes, rural residents, adolescents, migrants, and other vulnerable groups as requiring special attention. Consequently, the availability of reproductive services does not guarantee equal opportunity to use them.

ARTs particularly clearly demonstrate the gap between formal rights and the ability to realize them. In Kazakhstan, certain procedures are funded through the compulsory social health insurance system, but access depends on insurance status, medical criteria, and the type of treatment, and some services require personal expenses [64]. The funding criteria are also focused on a more favorable treatment prognosis, which may limit access for women with reduced reproductive potential [64]. Thus, equal reproductive rights do not mean equal opportunities to exercise them.

This trend extends beyond the scope of VRT. In Almaty, insurance coverage expands access but does not ensure equality in obstetric and gynecological care [67]. The ongoing demand for services that are already formally available for free also indicates a gap between their institutional availability and practical use, which is related to awareness and the complexity of navigating the healthcare system.

According to MICS data, 98.8% of women in Kazakhstan are aware of at least one method of contraception [65], but awareness does not equate to reproductive autonomy. Economic dependence, family control, gender asymmetry, and cultural norms may limit the use of formally available services [68]. Therefore, it is not only access to contraception that is important, but also the social opportunity to independently turn this access into a real choice.

This tension is particularly evident at the intersection of state reproductive policy with religious and traditional norms. Despite Kazakhstan's secular nature, reproductive decisions remain linked to the norms of marriage, motherhood, and female sexuality. Vaigorova and et al., show that abortion, while legally regulated, remains a socially sensitive choice shaped by religious, cultural, and family norms [63]. Therefore, religion does not operate in isolation but in conjunction with family authority, gender expectations, and economic dependence.

A particularly relevant mechanism is “uyat” a socially conditioned feeling of shame associated with behavior that is perceived as contradicting the expectations of the family or society. In a broader sociological sense, “uyat” (shame) can be viewed as a mechanism of internalizing social control: a woman does not necessarily have to face a direct prohibition if the expected condemnation from the family or community narrows the scope of permissible actions in advance. In this case, reproductive inequality is formed not only through restrictions on access to medical care but also through differences in the social freedom to take advantage of available assistance. Therefore, formally identical reproductive rights may have different practical value for women in different family and regulatory contexts.

Vaigorova et al., discussing teenage pregnancy and abortion, describe yat as stigmatization arising from the expected negative reaction from parents and relatives. According to the authors, the fear of condemnation may prevent teenagers from discussing the pregnancy, consulting with family members, or seeking medical help. Some teenagers avoided seeking medical care due to embarrassment, fear of judgment, or inability to pay for pregnancy termination services [63]. Vaigorova et al., also argue that abortion remains a socially sensitive and often taboo topic in Kazakhstan, and that parental involvement may exacerbate stigmatization rather than simply regulating access to abortion [63]. The authors link this pressure to traditional ideas about marriage and childbearing, showing that a family's reaction may push a woman to get married or keep the pregnancy instead of having an abortion, rather than doing what she herself wants. Thus, uyat (shame) is important not as a universal factor determining women's behavior, but as a mechanism that can increase the social cost of exercising reproductive choice recognized by law.

Overall, the relatively broad institutional recognition of reproductive rights in Kazakhstan does not mean their equal practical implementation. Access to formally available services still depends on financial and social resources, awareness, insurance status, family support, and a woman's autonomy. Therefore, reproductive inequality arises not only due to differences in health status but also in the ability to transform reproductive needs into a timely diagnosed and medically permissible need. In other words, inequality can emerge even after the right has been recognized and the medical service has been formally provided.

Summary Table 1 systematizes the identified gaps and implications, demonstrating the transition from describing inequalities to their operationalization in research design.

**Table 1:** A summary of the key research gaps and how this study addresses them in the context of intra-urban inequality and reproductive health (Almaty).

№	Research gap identified in the literature	What is missing (brief explanation)	How this study addresses the gap (contribution)
1	Insufficient localization of reproductive experience within a large city	The city is often viewed as a homogeneous context; everyday routes, neighborhood infrastructure, and local support networks are rarely analyzed as factors of actual access.	An intra-urban lens is used: The reproductive practices/attitudes of women in eight districts of Almaty are compared, linking the results to place of residence and urban context.
2	Insufficient integration of spatial and infrastructure indicators into sociological models	The analysis often relies on individual/household characteristics and formal service coverage, but does not capture the transaction costs of access (time, transport, queues, predictability, administrative barriers, social risks).	Inequality is interpreted through the mechanism of transaction costs: It is shown how “high coverage” can be combined with unequal actual access in different areas.
3	There is insufficient research on how household resources interact with the intra-urban opportunity structure.	Household resources and spatial context are often studied separately; it is not always tested whether differences persist after accounting for living conditions.	It is examined whether the links between household material well-being and reproductive indicators persist after accounting for age, education, employment, marital status, number of children, and urban embeddedness (district and duration of residence in Almaty).
4	Limited integration of multi-level social, institutional, and sociocultural contexts into the analysis of reproductive potential.	The use of reproductive healthcare services and family planning methods is often examined from the perspective of socio-economic indicators or service accessibility, without due consideration of how family norms, religious beliefs, gender expectations, and reproductive values influence the understanding of needs and available options.	From the perspective of ecological systems theory, household resources and reproductive practices are considered in the context of interacting conditions at the micro level, institutional and urban opportunities, as well as the broader sociocultural and political context.

***Note:*** The empirical base is a cross-sectional survey of women of reproductive age in Almaty (*N* = 934); reliability assessments of scales, contingency tables, and inter-district comparisons were used.

### 2.4 Research gaps and implications of the analysis of reproductive determinants

In the article, the logic of Opportunity Atlas is used not as a metaphor, but as a place-based design for measuring the urban heterogeneity of reproductive opportunities. The spatial level is operationalized through the respondent's administrative area of residence and a composite index of territorial conditions based on estimates of social infrastructure availability. This allows us to move from the general thesis “place matters” to verifiable measurements: A comparison of areas by reproductive opportunities and an analysis of how household income correlates with reproductive practices, taking into account the spatial context.

## 3 Methodology and data

The study has a cross-sectional design and is devoted to the analysis of socio-economic and spatial determinants of reproductive behavior of women of reproductive age in a large metropolis of Kazakhstan.

The theoretical and analytical framework of the study is based on the Capabilities Approach of Amartya Sen (interpreted by M. Nussbaum) [3], where reproductive health is considered as the distribution of real opportunities and reproductive autonomy, and not only as a medical outcome. The spatial component of inequality is operationalized through place-based logic inspired by Opportunity Atlas [34], [35], which is used as a diagnostic tool for territorial access gradients and the “field of opportunity” in an urban environment. In this work, Opportunity Atlas is used not as an independent theory of reproductive behavior, but as a methodological framework for spatial operationalization: The area of residence sets the context, and the composite index pROI acts as a perception-based proxy characteristic of a “place” reflecting the perceived infrastructural supportive environment.

Unlike the classic OA (administrative data, micro-geography), a survey proxy with district-level analysis is used here.

The field stage was held from spring-summer 2025 in Almaty, in eight administrative districts of the city: Alatau, Auezov, Almaly, Bostandyq, Zhetysu, Medeu, Nauryzbay and Turksib. The study was implemented by the research team.

The general population included women aged 18-49 years who permanently reside in Almaty. Inclusion criteria:

1. age 18-49 years;
2. experience of living in the city for at least five years;
3. having sexual experience.

Exclusion criteria:

1. Cognitive or linguistic limitations that prevent meaningful participation in the survey;
2. Current pregnancy with medical contraindications according to self - report;
3. Questionnaires that did not meet the data quality requirements for inclusion in the analytical set. This included duplicate questionnaires and entries containing significant errors in filling out or internally contradictory answers that prevented reliable interpretation of the respondent's data. Before statistical analysis, the questionnaires were checked for duplication, completeness, and logical consistency, and records that did not meet these quality requirements were excluded from the final set of analytical data.

Calculation of the minimum sample size.

The minimum sample size was determined using the standard Wald formula for estimating the proportion with simple random selection and a two-way 95% confidence interval:

$$n=\frac{Z_{1-\alpha /2}^{2}\;p\left(1-p\right)}{E^{2}}$$

where Z_(1-α/2) = 1.96 at α = 0.05, *p* = 0.50 (conservative estimate), E = 0.05. The calculation yields n ≈ 384. The correction for the final population was not applied, since the size of the general population is significantly higher than the calculated n. The actual volume of the survey was *n* = 934, which provides sufficient capacity for intergroup comparisons and multifactorial modeling.

The sample was formed using a stratified quota scheme and is classified as a disproportionate non-random sample, requiring caution when generalizing the results to the entire population of women in Almaty. Quotas were set for the city's administrative districts to ensure the representation of women of reproductive age permanently residing in Almaty and meeting the inclusion criteria. Data collection was conducted using a mixed approach combining online and offline recruitment. Of the total sample (*n* = 934), 630 respondents (67.5%) completed the questionnaire online, and 304 respondents (32.5%) were interviewed offline. Data collection in both modes was carried out in accordance with quotas set for a specific interviewer. The online data set was carried out by 17 interviewers, each of whom worked in accordance with an individually set quota and provided access to an independently completed questionnaire through certain communication channels. Offline data collection was conducted by six interviewers who also worked according to individually set quotas and recruited suitable respondents through direct contact in an urban environment. This combination was used to expand the reach of different social groups and reduce the risk of underrepresentation associated with using a single data collection method. To prevent duplicate responses, technical and substantive cleansing of the dataset was conducted, including checking for duplicate questionnaires, logically checking responses, and eliminating records with signs of duplicates or completion errors. The distribution of the sample by district ensured the inclusion of respondents from all eight administrative districts of Almaty, which allowed for inter-district comparisons; however, the obtained estimates should be interpreted as reflecting the identified associations and social gradients within the surveyed population, and not as strictly probabilistic parameters of the general population.

### 3.1 Instrumentation and operationalization of variables

The questionnaire consisted of 49 main questions: 14 socio–demographic questions and 35 substantive questions. Some of the questions included multiple items or sub-questions. The tool covered socio-demographic and economic characteristics, general and reproductive health, use of medical services and financial barriers, reproductive history and practice, awareness of reproductive health, as well as socio-cultural, environmental and economic factors related to reproductive behavior (Table 2).

**Table 2:** Conceptual structure of variables and their analytical significance.

Analytical level	Key variables /concepts	Theoretical and analytical interpretation
I. Reproductive behavior	The use of modern methods of contraception	It reflects the level of reproductive autonomy and rational control over fertility. It is an indicator of a woman's ability to realize her own reproductive intentions in the context of social constraints.
	Refusal of medical services for financial reasons	A symbol of structural inequality: Demonstrates how economic barriers transform into limitations of biographical opportunities and undermine trust in healthcare institutions.
	Reproductive health awareness	Cognitive measurement of reproductive chances. Health literacy acts as a mediator between education, income, and behavioral strategies.
	Postponing the birth of a child	Reproductive adaptation to external economic and institutional factors. Reflects the balance between structural constraints and personal agency.
II. Socio-economic determinants	Age, education, employment, income	Social and economic capital, which determines the level of opportunities and the degree of predictability of life strategies.
	Family status, presence of children	The components of the family structure that set cultural and normative expectations and reproductive roles in a gender context.
	Self-assessment of physical health	A perceptual indicator of bodily well-being; a key variable connecting the biological and social dimensions of health.
III. Spatial and contextual factors	Area and length of stay	Spatial rootedness, reflecting the degree of integration of women into the urban environment and access to infrastructural resources.
	Provision of the area with social infrastructure	An indicator of territorial justice. It demonstrates how the quality of the environment forms differences in real opportunities for health and family planning.
	Perception of environmental conditions	A cognitive marker of environmental vulnerability and risk perception; shows how the environment becomes part of a reproductive decision.

The table is not a technical list of variables, but an analytical framework linking individual, socio-economic, and spatial levels of analysis.

### 3.2 Skip processing and statistical analysis

Data gaps were processed according to the listwise deletion principle in multifactor models; in bivariate comparisons, only observations with valid responses for the analyzed pair of variables (pairwise deletion) were used. Weighting and multiple imputation were not used in this version of the analysis.

The statistical analysis included three consecutive stages:

1. Descriptive statistics (averages, medians, standard deviations, fractions, 95% confidence intervals);
2. Bivariate associations (χ2, t/F tests, Spearman and Pearson correlations);
3. Multifactorial modeling of binary and continuous outcomes (logistic and linear regression).

Multicollinearity and stability of estimates were tested; FDR was applied to families of tests. All calculations are performed in SPSS 23.

Due to the small number of clusters (J = 8), the main analysis is based on district fixed effects and/or cluster-robust SE by district in order to take into account immeasurable inter-district differences without unreliable random effects with a small J. Random intercept models are used only as robustness check; conclusions based on FE/cluster-robust specifications.

### 3.3 Place-based operationalization (opportunity atlas logic) and the pROI index

The place-based level is set by the area of residence (8 districts of Almaty). The context of the “field of opportunity” is measured through self-assessments of the accessibility/quality of everyday infrastructure: Education, clinics, trade/food, leisure/public spaces, sports, safety.

The composite index of perceived reproductive opportunities (pROI). The composite indicator of perceived infrastructure support was compiled based on six questions assessing the availability of educational institutions, medical facilities, retail and food service establishments, places for leisure and recreation, sports facilities, as well as the safety of the area. Responses were rated on a three-point scale (1 = fully available, 2 = partially available, 3 = unavailable). Responses marked as “difficult to answer” were considered missing. To calculate the pROI index, responses to six questions were reverse-coded so that higher values corresponded to more favorable conditions in terms of the perception of infrastructure opportunities. The responses were standardized using z-scores and averaged with equal weighting coefficients. Thus, higher pROI index values indicate a more favorable perceived infrastructure environment in terms of the availability of local services and the safety of the area.

The internal consistency of the six-question scale was assessed using Cronbach's alpha coefficient. The reliability analysis was conducted based on complete data for all six questions after responses marked as “difficult to answer” were deemed missing. The scale demonstrated good internal consistency (Cronbach's alpha = 0.814; standardized alpha coefficient = 0.815; *n* = 780). The corrected correlations between each question and the total score ranged from 0.535 to 0.605. Cronbach's alpha, with one item removed, ranged from 0.777 to 0.793. Thus, removing any single item did not improve the overall internal consistency of the methodology.

pROI is introduced as a contextual (meso) predictor in models of reproductive outcomes (financial refusals of care, access to paid medicine, savings on contraception/postponement/termination, actual use of contraception). Income (personal + subjective well-being of the household) is checked for the sustainability of the effect under the control of pROI; in multi-level specifications, district = cluster, pROI = measurable characteristic of a place in the logic of Opportunity Atlas.

The dimensional structure of the six-item questionnaire was further assessed using exploratory factor analysis (EFA) with principal axis factoring (PAF) based on a correlation matrix. Responses marked as “difficult to answer” were considered missing, and the analysis was carried out using data for all six items. The adequacy of the sample and the possibility of factorization were assessed using the Kaiser–Meyer–Olkin (KMO) criterion and the Bartlett sphericity test. The number of retained factors was determined using the criterion that the eigenvalue must be greater than one, as well as through visual analysis of the scree plot. To assess the contribution of each item to the identified factor structure, factor loadings and extracted communality were examined. Rotation was not applied, as the extraction resulted in a single factor.

## 4 Results

### 4.1 Socio-demographic profile of the study participants

In the sample of 18-49 years old: The average age is 32.8 (SD = 9.7), the median is 32 (IQR 24-41); the largest groups 25-34 (31.8%), 35-44 (25.8%) and 18-24 (25.7%). This age-related “mid-life cycle” is important methodologically, it captures not the adolescent norm, but the decisions and limitations experienced in a real family and work biography. 65.0% are married/in partnership, 17.5% are divorced; 73.9% have children, most often two (29.8%). This makes the selection based on experience rather than just attitudes. The entire socio-demographic profile of the respondents is highlighted and shown in Table 3.

**Table 3:** Socio-demographic profile of the participants (*n* = 934).

Indicator	Categories	n	%
Age	Average (SD); Median (IQR)		32.8 (9.7); 32 (24–41)
	<18	3	0.3
	18–24	240	25.7
	25–34	297	31.8
	35–44	241	25.8
	45–49	153	16.4
Education	Incomplete secondary education	29	3.1
	General average	101	10.8
	College/Vocational school	251	26.9
	Bachelor	388	41.5
	Master	124	13.3
	Studying for a PhD	22	2.4
	PhD and higher	19	2
Employment	Private sector (hiring)	233	24.9
	Budget organization (hiring)	154	16.5
	Civil Service	147	15.7
	Self-employed/Sole proprietor	199	21.3
	Student	71	7.6
	On nursing leave/housewife	67	7.2
	Temporarily out of service	37	4
	I don't work for health reasons	15	1.6
	The pensioner	11	1.2
Family status	Married	469	50.2
	Unregistered marriage	138	14.8
	Divorced	163	17.5
	Widow	36	3.9
	I've never been married.	128	13.7
Children	No children	244	26.1
	1 child	174	18.6
	2 children	278	29.8
	3 children	173	18.5
	4+ children	65	6.9
Household welfare	We can buy a car/apartment	96	10.3
	Enough for everything except very expensive	472	50.5
	There is enough for food / clothes, the goods are difficult to last. Use	243	26
	Enough for food, clothes are difficult	82	8.8
	Not even enough for food	9	1
	I find it difficult to answer	27	2.9
Residential area	Alatau	113	12.1
	Almaly	151	16.2
	Auezov	144	15.4
	Bostandyq	171	18.3
	Zhetisu	98	10.5
	Medeu	91	9.7
	Turksib	97	10.4
	Nauryzbay	64	6.9

#### 4.1.1 Personal income of the study participants

The personal income distribution in the sample exhibits a pronounced right-hand asymmetry: A small proportion of high values ”raises” the mean, so the average income (282,513 tenge; USD 567.28) is significantly higher than the median (230,000 tenge; USD 461.84). This is a typical configuration for urban income data, where different economic trajectories coexist; therefore, when interpreting the “typical” level, it is more accurate to rely on the median and interquartile range.

If we restrict the sample to participants with income >0 (*n* = 896), the key parameters remain virtually unchanged: The median rises to 240,000 tenge (USD 481.92), and the mean to 289,133 tenge (USD 580.55), confirming that the asymmetry is driven by the upper segment of the distribution. At the level of the most frequent values, a concentration around “round” amounts is noticeable: 300,000 (602.40 USD), 500,000 (1004.00 USD), 200,000 (401.60 USD), 150,000 (301.20 USD) and 400,000 tenge (803.20 USD), which further indicates the “anchoring” of self-assessments of income to socially recognizable thresholds (Table 4).

**Table 4:**
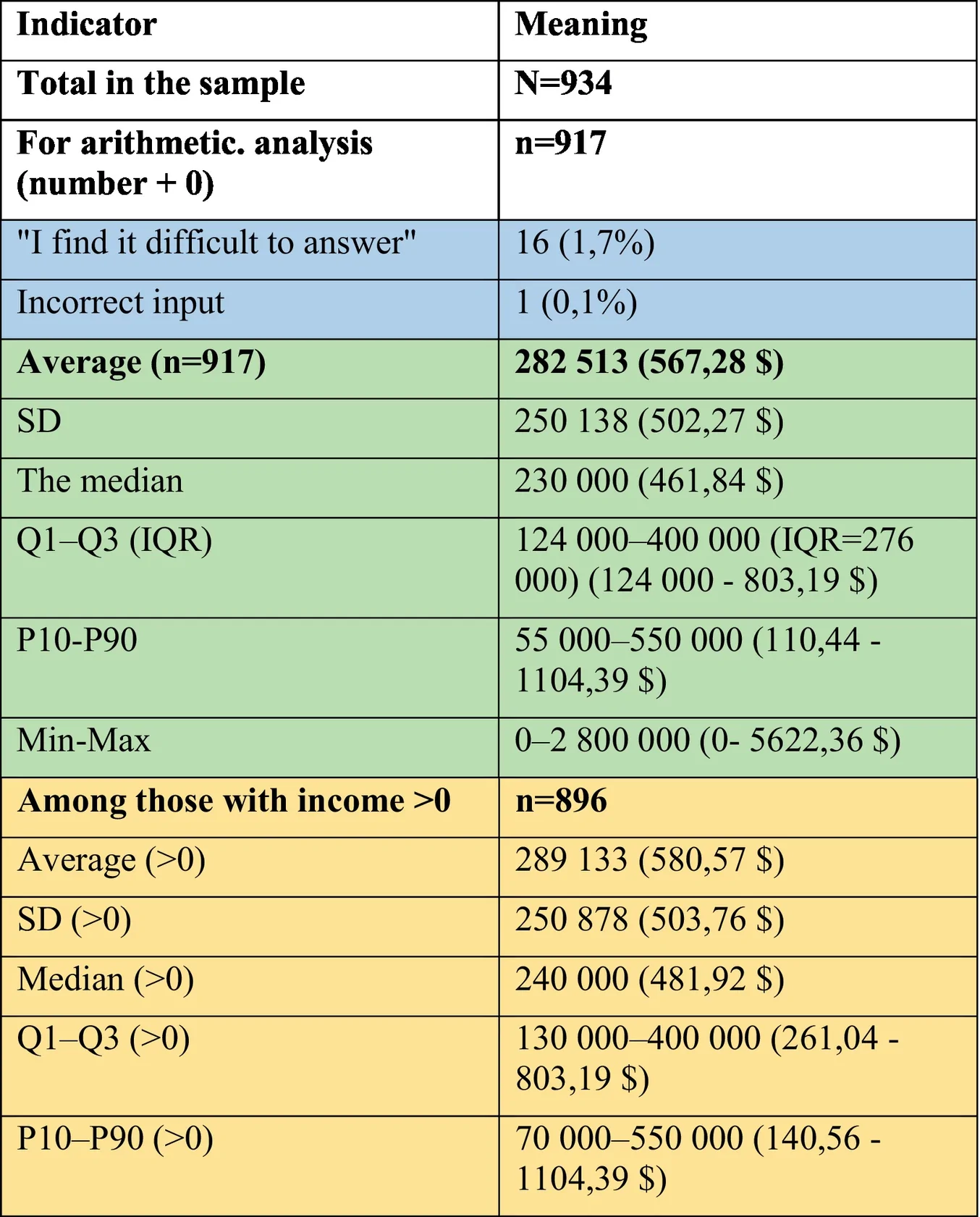
Personal monthly income, tenge (in arithmetic terms), 1 $ = 498.01 tenge.

**Note.***n* = 917 participants with a valid numerical value of their monthly personal income (including 0); 16 “I find it difficult to answer” responses and 1 incorrect input were excluded from arithmetic calculations. Indicators “>0” are calculated among participants with incomes above zero. Q1–Q3 is the 25th-75th percentile (IQR = Q3–Q1); P10–P90 is the 10th-90th percentile.

The sample was characterized by pronounced ethnic and religious heterogeneity. Of the 934 women surveyed, 49.7% (*n* = 464) identified themselves as Kazakh, 21.1% (*n* = 197) as Russian, and 10.4% (*n* = 97) as Uyghur. This was followed by Korean 6.0% (*n* = 56), Tatar 4.9% (*n* = 46), Uzbek 3.7% (*n* = 35), and Kyrgyz 1.4% (*n* = 13). Representatives of other ethnic groups accounted for less than 1% of the sample each.

In terms of religious affiliation, the largest group consisted of women who identified as Muslim — 70.3% (*n* = 657). 10.3% (*n* = 96) indicated Orthodoxy, 3.6% (*n* = 34) indicated Buddhism, and 2.4% (*n* = 22) indicated Catholicism. 4.4% (*n* = 41) identified as agnostic, and 1.5% (*n* = 33) identified as atheist; a few respondents indicated a secular stance (0.2%; *n* = 2) or deism (0.3%; *n* = 3). 2.0% (*n* = 19) refused to answer the question about religious affiliation, and another 2.9% (*n* = 27) found it difficult to answer. Thus, along with the predominance of Islamic self-identification, the sample included women of various religious and non-religious orientations.

#### 4.1.2 Sociocultural conditions of reproductive choice

The results show that family traditions still influence reproductive decisions, but they are more often combined with individual autonomy. 41.5% of the respondents (*n* = 388) indicated that family traditions are important to them, but decisions are made taking into account their own preferences and modern conditions; 26.7% (*n* = 249) defined decisions about children and contraception as their own choice. At the same time, 12.7% (*n* = 119) associated early marriage and childbirth with family values, and 10.7% (*n* = 100) reported socialization in a conservative environment where discussion of reproductive issues was limited.

The sources of pressure turned out to be diverse. 13.5% of women (*n* = 126) reported pressure from their partner; 12.6% (*n* = 118) from their employer or colleagues due to pregnancy and maternity leave; 8.8% (*n* = 82) from family, relatives, or society; and 6.2% (*n* = 58) from religious and cultural norms. At the same time, 21.6% (*n* = 202) reported complete autonomy in reproductive decisions, and 10.4% (*n* = 97) reported open family discussion without pressure.

The descriptive differences between the largest religious groups were moderate. Among women who identified as Muslim, 42.2% combined family traditions with personal choice; among Orthodox Christians, 43.8%; 25.1% and 25.0%, respectively, reported exclusively autonomous choice. Direct religious and cultural pressure was noted by 6.8% and 5.2%, respectively. This does not allow us to consider religious affiliation as a simple marker of traditional reproductive behavior.

Overall, the results reflect not the disappearance of traditional norms, but their restructuring within a more individualized decision-making model. For the modern urban environment of Kazakhstan, reproductive autonomy is formed simultaneously in the family, in partnerships, and in the workplace; therefore, its limitations cannot be reduced solely to religion or “traditionality”. A more accurate model is one in which a woman coordinates her own reproductive plans with several competing systems of expectations.

### 4.2 Results: spatial differentiation of the perception of urban infrastructure depending on the type of housing and the residential area

An assessment of six aspects of perceived urban infrastructure: Educational institutions, medical facilities, retail and food service businesses, leisure and entertainment venues, sports facilities, and safety in the area revealed a clear hierarchy: basic social infrastructure is perceived as more accessible than infrastructure related to the daily quality of life. At the level of the entire sample, minimal deficits are recorded in the domains of education and primary health care, while the maximum deficits are in safety, as well as in sports and leisure opportunities. The proportion of responses “not provided at all” consistently increases from education (8.2%) and polyclinics (9.3%) to trade and service infrastructure (14.7%), leisure (18.9%), sports (18.9%) and reaches a peak in safety (20.3%). In a social sense, this difference is fundamental: The respondents separate the accessibility of institutions (where one can get to) from the viability of the environment (in which one can live peacefully). It is the second layer of security, free time, movement, and physical activity that creates a sense of vulnerability and limits real urban opportunities.

The key conclusion is that the observed spatial differentiation is not so much related to the boundaries of administrative districts as it is to the trajectories of housing movement. The type of residence is not just a household characteristic, but an indicator of how a household is embedded in a city: How stable its resource contour is, how predictable localization, transport, environment and the right to a quiet daily routine are. At this level, the differences become sharp: Tenants are much more likely to have an extreme assessment of “not provided at all” specifically for high-quality environmental domains. Compared to living in your own apartment/house, living in a rented apartment is associated with a threefold increase in the deficit of trade and service infrastructure (24.1% vs 7.0%) and an approximately twofold increase in deficits in sports and safety (25.1–26.1% vs 12.8–13.3%). In other words, renting in a megalopolis functions as a form of spatial precarity: It often places a woman in a part of the city where everything is technically there, but everyday life is difficult and unsafe, there is nowhere to go out, nowhere to recover, and barriers to regular bodily activity are higher.

The most vulnerable accommodation formats (rented rooms, dormitories, etc.) demonstrate maximum levels of security deficits. Although small n's should be considered for individual categories, the response profile itself is conceptually important: The less control over housing, the less control over the environment and the higher the perceived risk. This is especially significant from a gender perspective: for women, security is not an abstract indicator of urban policy, but an everyday condition for mobility, access to services, employment regimes, and the ability to plan a life without constant anxiety allowances.

The administrative area of residence sets the differences, but they are smoother: There are inter-district gradations in safety and sports, but they are less contrasting than the differences between property and rent. In terms of urban sociology, this means that administrative geography captures the background, while the housing form more accurately reveals structural inequalities at the level of everyday life at the yard level, route, street lighting, service density, and cost of access in time and effort.

Six aspects of perceived urban infrastructure were positively correlated with each other, with the correlation between individual aspects ranging from 0.327 to 0.517. The strongest association was observed between the perceived availability of educational and medical facilities (*r* = 0.517). This pattern indicates that perceived infrastructure shortcomings typically manifest in several aspects simultaneously, rather than in isolation, which suggests a cumulative nature of limitations in the everyday urban environment.

As a result, the results describe not just the distribution of infrastructure, but the mechanism of urban stratification: In a megalopolis, basic institutions remain relatively accessible, but the quality of the environment, safety, leisure and sports, becomes the very dimension where inequality manifests itself more harshly and closer to the body. It is here that the housing trajectory (ownership vs rent) turns into a hidden geography of access to urban opportunities and, more broadly, to the reproduction of health and life strategies (Table 5, Table 6).

**Table 5:** Type of housing and perceived deficit of urban infrastructure: the share of respondents who noted that the infrastructure is “completely absent,” %. (Percentage figures are calculated for each housing category; the areas with the greatest differentiation are presented.)

Type of accommodation	n	Trade/catering	Sport	Safety
In my own apartment/house	445	7	13.3	12.8
In a rented apartment/house	307	24.1	25.1	26.1
In a rented room	43	18.6	23.3	34.9
In the apartment of relatives	92	12	20.7	25
Other forms of accommodation*	42	31	28.6	35.7

***Note:*** Combined (small n): friends' apartment / employer's housing / hostel / hostel.

**Table 6:** Residential area and urban environment deficit: Percentage of “not provided at all,” %. (within the area; domains with the greatest contrasts are shown).

District	n	Trade/catering	Sport	Safety
Alatau	113	17.7	26.5	22.1
Almaly	151	8.6	12.6	16.6
Auezov	144	9.7	16.7	16
Bostandyq	171	12.3	11.1	17.5
Zhetisu	98	21.4	27.6	30.6
Medeu	91	18.7	19.8	22
Turksib	97	17.5	25.8	25.8
Nauryzbay	64	21.9	23.4	18.8

***Note:*** The shares are calculated as the percentage of responses “3 - not provided at all” within the corresponding group. The category “difficult to answer” is saved in the data, but it is not included in the calculation of the deficit share.

### 4.3 Results: spatial and socio-economic differences in access to services and reproductive practices

The analysis was performed on the initial array of survey data (*N* = 934) without the use of weighting factors. All the cross-tables took into account only observations with valid answers for the analyzed pair of indicators (pairwise deletion), so the final sample size varied depending on the question. The most complete data were obtained for indicators of financial constraints on access to reproductive health services, the current method of contraception, and the reasons for postponing the birth of a child (valid N ≈ 921-929). Questions with clarifying/additional answers on contraception had a significantly lower proportion of valid observations (*n* = 382 and *n* = 180), which should be taken into account when interpreting: In such tables, the sparsity of categories is higher and the statistical power of tests is lower (Table 7).

**Table 7:** Valid observations on key indicators.

Indicator	Brief content	Valid N	Valid ones, %	Omissions N	Omissions, %
Financial restrictions on access to reproductive health services	Refusals/restrictions on receiving services for financial reasons	929	99.5	5	0.5
Current method of contraception	The method used most often at the time of the survey	926	99.1	8	0.9
Contraception (additional/clarifying answer)	Additional item on methods of contraception	382	40.9	552	59.1
Life-long contraceptive experience	The method most often used in life	180	19.3	754	80.7
Postponing the birth of a child	Reasons for postponement due to external factors	921	98.6	13	1.4

***Note:*** The high proportion of omissions on two indicators of contraception is related to the structure of the instrument (clarifying/additional responses) and reduces the statistical power of the corresponding cross-tables; therefore, these results are interpreted as auxiliary.

### 4.4 Results: spatial differences: residential area

The analysis shows that the area of residence is associated with the experience of financial constraints when applying for family planning and reproductive health services (χ2(28) = 44.940, *p* = 0.022), but the effect is small (Kramer's V = 0.110). This is an important nuance for a megalopolis: Administrative geography records the differences, but does not fully explain them. The inter-district contrast is evident not only in the fact that some women refuse services “regularly/in some cases,” but also in the proportion of responses “I do not apply for services.” The latter is particularly meaningful: In large cities, inequality is often expressed not in a bad area as such, but in different modes of inclusion in the service system, some maintain contact with services, others drop out of it, because the price of access consists of money, time, logistics and psychological costs.

Against this background, the district does not act as a stable predictor of everyday reproductive practices: The association of the district with the current method of contraception and with the reasons for postponing the birth of a child has not been statistically confirmed (*p* > 0.05). This looks like a typical pattern of megalopolis normalization: Practices and motives (contraception, procrastination) are more shaped by general urban conditions, the labor market, and family strategies than by administrative boundaries. The borderline result of the contraceptive experience “during life” (*p* = 0.084) with a sparse table and a small valid N (180) should be interpreted as an exploratory signal, without strengthening the interpretation.

The final conclusion: The area of residence in Almaty works as a soft contextual factor that can highlight differences in financial barriers and even in the likelihood of seeking services, but does not directly determine the choice of contraception or the reasons for postponing birth. The key thing to explain is not “where you live” in the administrative sense, but what resources you have and how your trajectory of access to services in a city with a high cost of time and daily logistics is arranged (Table 8).

**Table 8:**
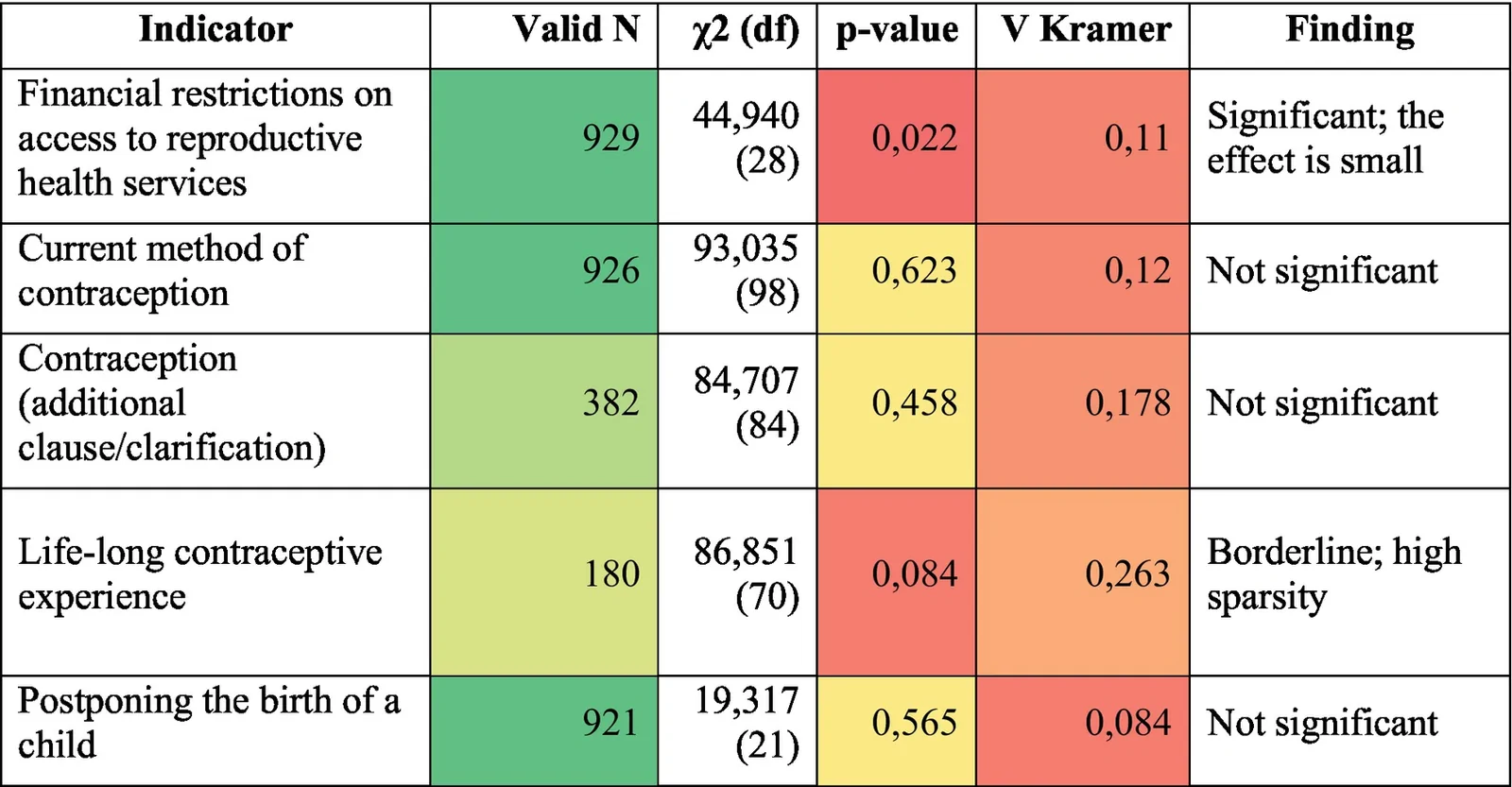
Connections with the area of residence: Significance and strength of effects. The color coding shows the completeness of the data (green – a high proportion of valid responses; yellow – low/limited), statistical significance (red – *p* < 0.05; orange – 0.05 ≤ *p* < 0.10; yellow – *p* ≥ 0.10) and the strength of the Kramer V connection (the richer the shade, the stronger the effect).

### 4.5 Results: socio-economic gradient: household's material well-being

The relationship between the household's material well-being and financial constraints on access to reproductive health services is pronounced and highly significant (χ2(20) = 101.194, *p* < 0.001), and the strength of the effect is noticeably higher than that of the territorial factor (V = 0.165). In the logic of a megalopolis, this means a simple thing: The main barrier is not where you live, but which resource contour holds you. In this sense, Almaty works as an access market: Formally, services may be available, but in fact, entry into them is regulated by money, time, and cost predictability.

The key observation is not only in the failure rate. The social meaning is revealed in the growth of the “don't apply” strategy among groups with lower well-being. This does not seem like a one-time choice, but a habit of limitation: When the expectation of expenses, the experience of inconvenience, doubts about quality and fatigue from the system gradually transfer a woman from the preventive regime to the regime of forced silent patience. This is critical for reproductive health: Exclusion occurs not through prohibition, but through self-excision, which statistics usually catch worse than outright refusals.

Material well-being is related to the structure of current contraceptive practices (χ2(70) = 143,805, *p* < 0.001; V = 0.176). This is not a taste for methods, but a social architecture of choice: well-being shifts the entire profile of practices, from access to counseling and regular use to the likelihood of not using or falling out of the modern family planning circuit.

The important conclusion here is that contraception in a megalopolis is not only a medical decision, but an institutional habit that requires resources (information, time, trust in doctors, money for a method and monitoring). Therefore, the differences appear systematically, rather than point-by-point in rare categories: Economic status sets not just one option, but a range of real possibilities.

The association of well-being with the reasons for postponing the birth of a child is significant, but weaker in effect (χ2(15) = 28.230, *p* = 0.020; V = 0.101), which empirically confirms that postponement is not a monocausal financial history. However, the material factor remains a stable part of the explanation: It does not work as the only reason, but as a background of risk that makes other reasons (work/study, health, relationships) more expensive and less manageable.

In this sense, the metropolis produces a paradox: The higher the cost of a mistake (loss of income, housing, child care), the more rational the delay looks, and the stronger the stratification between those who can plan and those who have to wait. Postponement is becoming not a fashion or an individual whim, but a strategy for managing uncertainty, where money sets the boundaries of what is possible.

Taken together, the results show that material well-being in Almaty is not just a social background, but a functional equivalent of infrastructure: It determines whether a woman will be included in the preventive circuit of reproductive health or will find herself in a mode of forced deferred treatment and limited choice (Table 9).

**Table 9:** Results of tests related to household material well-being.

Indicator	Valid N	χ2 (df)	p-value	V Kramer	Finding
Financial restrictions on access to reproductive health services	929	101,194 (20)	<0.001	0.165	Significant; pronounced socio-economic gradient
Current method of contraception	926	143,805 (70)	<0.001	0.176	Significant; social profile differentiation
Contraception (additional clause/clarification)	382	78,074 (60)	0.058	0.202	Borderline; limited capacity
Life-long contraceptive experience	180	56,728 (50)	0.239	0.251	Insignificant; small N and sparsity
Postponing the birth of a child	921	28,230 (15)	0.02	0.101	Significant; small effect

### 4.6 pROI and “district gradients”: district profiles of financial barriers and birth postponement

In the logic of Opportunity Atlas, the area of residence is considered as an approximate indicator of the contextual environment of a megalopolis, a combination of accessibility of services, everyday infrastructure, local “cost of treatment” (money, time, transport and organizational load) and social resources that make it easier or more difficult to receive assistance. Two outcomes were used as observed behavioral indicators: The experience of financial refusals from medical services, including family planning and reproductive health, and postponing the birth of a child due to external circumstances. At the descriptive level, moderate heterogeneity is revealed between the eight districts of Almaty: The range of average values is 0.42 points for financial constraints (2.67–3.09) and 0.21 points for postponing birth (2.77–2.98). These differences should be interpreted as differences in the overall configuration of responses, rather than as an accurate “rating” of districts.

The indicator of financial constraints has an ordinal rather than an interval nature (the response options reflect gradations from regular refusals to the absence of restrictions and non-use of services). Therefore, the average values in Table 10 are used as a compact atlas indicator of the distribution bias (directional index), which allows comparing areas in terms of the direction and overall intensity of barriers, rather than measuring the “distances” between categories as equal. As part of the pROI approximation, such an indicator is appropriate because it provides comparability of districts on a single scale and visualizes how the distribution of responses in each district is shifted from regular financial refusals to more prosperous access regimes.

**Table 10:** “Atlas descriptives”: Regional averages, SE and 95% CI for two outcomes. (MEANS by area of residence; N = 934; S15 N = 929, S28 N = 921).

Panel A. Financial restrictions on access to reproductive health services (higher Mean → distribution further away from regular financial refusals).						
Residential area	N	Mean	SD	SE (mean)	95% CI(mean)	Rank*
Alatau	113	2.67	1.206	0.113	[2.45; 2.89]	8
Almaly	151	2.92	1.158	0.094	[2.74; 3.10]	7
Auezov	144	2.97	1.167	0.097	[2.78; 3.16]	6
Zhetisu	98	3.00	1.218	0.123	[2.76; 3.24]	4–5
Medeu	91	3.00	1.135	0.119	[2.77; 3.23]	4–5
Turksib	97	3.04	1.163	0.118	[2.81; 3.27]	3
Bostandyq	171	3.05	1.129	0.086	[2.88; 3.22]	2
Nauryzbay	64	3.09	1.218	0.152	[2.79; 3.39]	1
Total	929	2.96	1.171	0.038	[2.89; 3.03]	–


Panel B. Postponement of the birth of a child due to external factors. (categories of reasons; Mean reflects the profile of dominant reasons, not “more/less”)						
Residential area	N	Mean	SD	SE (mean)	95% CI(mean)	Profile (rank by Mean)
Nauryzbay	64	2.77	1.080	0.135	[2.51; 3.04]	8
Turksib	94	2.80	1.113	0.115	[2.57; 3.02]	7
Almaly	151	2.82	1.155	0.094	[2.64; 3.00]	5–6
Auezov	142	2.82	1.157	0.097	[2.63; 3.01]	5–6
Zhetisu	98	2.86	1.103	0.111	[2.64; 3.08]	4
Bostandyq	169	2.90	1.111	0.085	[2.73; 3.07]	3
Alatau	112	2.96	1.170	0.111	[2.74; 3.18]	2
Medeu	91	2.98	1.043	0.109	[2.77; 3.20]	1
**Total**	**921**	**2.87**	**1.121**	**0.037**	**[2.80; 2.94]**	–

***Panel A: Rank: 1*** = the most “favorable” profile for this index (the average is further away from regular failures).

Table 10 shows the district averages, standard errors, and 95% confidence intervals for both outcomes (*N* = 934; valid answers: Financial constraints – 929, birth delay – 921). Panel 10 A demonstrates the profile of financial barriers: A higher average value reflects the distribution of responses, in which regular financial refusals are less common and less severe forms of restrictions or the absence of such restrictions are more often recorded.

In terms of pROI-logic (the area as a context of risk and accessibility), the differences between the districts of Almaty in terms of financial constraints do not look dramatic in scale, but stable in direction. The most vulnerable profile is recorded in Alatau region (average value 2.67), while the most favorable in Nauryzbai (3.09) and Bostandyq (3.05). These gaps are small, but it is precisely this “thin gradient” that is typical for a megalopolis: Formally, the city offers a similar set of services to almost everyone, but real accessibility breaks down into different modes, in some cases medical care remains in the planning and prevention area, and in others it often turns into a “residual service” that is postponed., are reduced or avoided due to the price of treatment and associated costs (time, travel, recording, quality uncertainty). Therefore, it is appropriate to use regional averages here not as an absolute “better/worse” rating, but as an atlas navigation through the vulnerability context, where the probability of a financial barrier and loss of contact with the system is higher.

At the same time, it is important not to overload geography with what is not in the data: differences between areas do not mean that the reason lies in the area itself. Rather, the district acts as a marker of an urban environment where the cost of access to assistance, monetary and non-monetary, is different. In this sense, cartography shows not the territorial fate, but the local conditions of inclusion in services.

For the indicator of postponement of the birth of a child, regional differences should be interpreted even more carefully. Here, the scale captures the type of external factor (finances, work/study, health, or lack of such situations), rather than the linear intensity of “more/less procrastination.” Therefore, the correct conclusion is not to rank areas by the level of postponement, but that the spatial context can change the structure of the reasons through which women explain postponement, and this requires an analysis of the distributions by category, rather than reading the average values as a level.

### 4.7 Main models: NOMREG for “financial access restrictions”

To separate the neighborhood effect itself from differences in population composition (that is, from the fact that groups with different ages, education, and resources live in different neighborhoods), we evaluated a multinomial logistic regression for the outcome of “financial constraints in obtaining medical services, including family planning and reproductive health.”

The model included: The area of residence (as a contextual factor), as well as key socio-demographic characteristics that potentially compositionally explain the differences: Education level, family status, presence/number of children, employment; additionally, the age and material well-being of the household were controlled. The position “I find it difficult to answer” was set as the base category of the outcome, and the Nauryzbai district was set as the reference territory.

This design makes it possible to interpret district coefficients not as a simple map of differences, but as a contextual association with comparable individual characteristics: That is, it shows whether differences remain between districts when comparing women of the same age and comparable social profile in education, marital status, parenthood, employment, and household resources (Table 11).

**Table 11:** Model fitting and effects contribution (Likelihood-ratio tests).

Effect	LRT p-value	Interpretation
Household's material well-being	<0.001	It is most consistently related to the structure of the S15 responses
Education level	<0.001	marked stratification of access/treatment practices
Area of residence	0.088	contextual association on the border of significance as a whole, but manifests itself in individual contrasts
Availability/number of children	0.069	borderline; estimates are unstable due to sparsity of categories
Occupation/employment	0.060	borderline; possible sensitivity to category enlargement
Age	0.228	In this specification, the contribution is weaker
Family status	0.227	The overall contribution is weaker, but “local” differences in individual contrasts are possible.

Model fit: −2LL(final) = 2528.253, χ^2^ = 253.560, df = 136, p < 0.001.

Pseudo-R^2^: Cox&Snell = 0.239, Nagelkerke = 0.251, McFadden = 0.091.

Analyzing the results that SPSS gave us about the singularities of the Hesse matrix and overflow for some of the parameters (especially in the “presence/number of children” block). This is a typical sign of quasi-complete separation with a large number of categories and rare combinations. Consequently, individual coefficients (and their CI) can be considered as potentially unstable, and conclusions can be formulated at the level of association patterns and contribution tests (LRT), supplementing them with robust checks.

#### 4.7.1 “Model 1” (income → outcome): the core of associations of household material well-being with financial failures

Even in the full model, which simultaneously takes into account the area of residence and key socio-demographic characteristics, the household's financial well-being retains an independent and statistically stable relationship with the likelihood of financial constraints when applying for reproductive health services (likelihood ratio test, *p* < 0.001). It is important that we are not talking about a single triggered coefficient: The welfare effect manifests itself in several outcome categories, forming a reproducible gradient as resource availability decreases, the likelihood of access modes associated with financial barriers and loss of regular use of services increases. This result supports the interpretation that in a megalopolis, the key mechanism of access inequality is determined primarily by the resource stability of the household, and territorial differences act more as a contextual background that enhances or weakens the effect of this resource factor (Table 12).

**Table 12:** Material well-being and financial barriers to access: OR from the multinomial model. (Reference category of the result: “I find it difficult to answer”).

Response category for S15 (vs ref = “not sure”)	B – material well-being	p	OR = Exp(B)	95% CI OR
“regularly refused”	−0.027	0.012	0.973	[0.952; 0.994]
“in individual cases”	−0.024	<0.001	0.977	[0.965; 0.989]
“no financial restrictions”	−0.026	<0.001	0.975	[0.963; 0.987]
“I do not seek care”	−0.024	0.001	0.976	[0.962; 0.991]

In this specification, odds ratios are interpreted strictly relative to the basic category of the “I find it difficult to answer” response. Therefore, the task of this block is not to prove causality, but to accurately record three things: (i) material well-being is consistently linked to the structure of responses about financial barriers to access, (ii) the effect does not manifest itself at one point, but is reproduced in several outcome categories, and (iii) for a more intuitive and applied interpretation, a robustness check is necessary with An alternative basic category is, first of all, “there are no financial restrictions.” This is a standard sensitivity procedure to ensure that conclusions are independent of the selected reference.

#### 4.7.2 The model (base): household's material well-being and financial refusals from reproductive health services

The multinomial logistic model was evaluated for self-assessment of financial constraints when applying for medical services related to family planning and reproductive health (response options: regularly / in some cases / there are no financial constraints / I do not apply / I find it difficult to answer). Even in the full specification, which simultaneously takes into account the area of residence and the main socio-demographic characteristics (age, education, marital status, having children, and employment), the household's financial well-being remains the most stable factor associated with differences in response structure (likelihood ratio test for the welfare effect: *p* < 0.001).

Meaningfully, this means the following: In a megalopolis, the financial barrier manifests itself not only as a direct refusal to have money, but also as a broader resource mode of access, when some women are forced to reduce the intensity of requests or drop out of regular use of services. It is important that the relationship of well-being is observed in several key outcome categories compared to the base group. I find it difficult to answer, which indicates a systematic effect rather than a single local contrast. In this sense, the results support the interpretation of financial constraints as a structural mechanism of access inequality, which persists even after taking into account the territorial context and socio-demographic profile.

Table 13 summarizes the odds ratios (OR) reflecting the association of a household's material well-being with the likelihood of belonging to different financial restriction regimes compared to the basic category of “I find it difficult to answer”.

**Table 13:** Household material well-being and financial barriers to access: OR from the multinomial model (base: “I find it difficult to answer”).

Outcome category (financial constraints) vs reference (“I find it difficult to answer”)	B – material well-being	p	OR	95% CI OR
“regularly refused”	−0.027	0.012	0.973	[0.952; 0.994]
“in some cases”	−0.024	<0.001	0.977	[0.965; 0.989]
“there are no financial restrictions”	−0.026	<0.001	0.975	[0.963; 0.987]
“I'm not applying”	−0.024	0.001	0.976	[0.962; 0.991]

#### 4.7.3 Area: weak overall effect, but individual contrasts are visible

Even after taking into account age, education, marital status, children, employment, and financial situation, the area of residence remains associated with financial barriers to access, but at the borderline level (*p* = 0.088). This means that the district does not set a single ladder for better or worse, but is manifested by point differences, not the level, but the mode of access changes (they regularly refuse/ sometimes refuse/ there are no restrictions/ do not apply at all).

The most noticeable signal in the response was “regularly refused for financial reasons”: Compared to the Nauryzbai district, the probability of such an answer is higher in

1. Alatau (OR = 8.44; *p* = 0.014)
2. Almaly (OR = 7.41; *p* = 0.021).

Also, for the answer “there are no financial restrictions” in some areas, the probability is higher than in Nauryzbay (OR about 2.7–3.6; significant for Almaly, Turksib, Auezov and Bostandyq). Important: These estimates should be read carefully, because the confidence intervals are wide in places, and the model warns of instability (sparse cells). Therefore, it is correct to talk about contextual associations, rather than causality.

The key interpretation for a megalopolis is that the area does not affect the background as a better/worse background, but as a switch of practices. In some places, a regime of regular financial failures occurs more often, in others there is no restriction mode, and this is already important for the atlas logic of the pROI.

Education provides the most consistent contribution to explaining differences (*p* < 0.001). This means: Financial barriers, not just geography, but social stratification. However, it is better not to inflate the specific coefficients for education in the current model setup, because the PhD reference category and higher are rare, and sparsity increases the sensitivity of estimates. Therefore, it is correct to draw a general conclusion: The level of education is related to which access mode a woman gets into, and the exact size of the effects must be confirmed in sustainability checks (for example, by enlarging the categories of education).

### 4.8 Perceived reproductive opportunity index: how we “collected” the opportunity index and what it showed in the metropolis

The pROI indicator combines six aspects of the perception of urban infrastructure: educational institutions, medical facilities, retail trade and food service, leisure and recreation, sports facilities, and safety in the residential area. It is interpreted as a perception-based indicator of everyday infrastructure conditions that affect reproductive capacity.

#### 4.8.1 The six-item scale demonstrated good internal consistency

Reliability analysis based on complete responses to all six items related to infrastructure showed that Cronbach's alpha was 0.814 (standardized Cronbach's alpha = 0.815; *n* = 780). The corrected correlations between each item and the total score on the scale ranged from 0.535 to 0.605, indicating a significant contribution of each component to the overall assessment. Cronbach's alpha, when individual items were excluded, ranged from 0.777 to 0.793; thus, the exclusion of any individual item did not increase the overall reliability of the scale. The correlations between the items ranged from 0.327 to 0.517, which indicates the presence of stable positive relationships between the six aspects of the perception of urban infrastructure. Taken together, these results confirm the internal consistency of the six-item scale.

Exploratory factor analysis confirmed the unidimensional structure of the six-item questionnaire. The Kaiser–Meyer–Olkin coefficient showed good adequacy of the sample (KMO = 0.852), and Bartlett's sphericity test was statistically significant (χ^2^(15) = 1317.358, *p* < 0.001), which confirms the suitability of the correlation matrix for factor analysis. Factor analysis using the principal component method allowed us to identify one factor in accordance with the criterion “eigenvalue greater than one” (initial eigenvalue = 3.116), while the scree plot showed a clear break after the first factor was identified. The first factor explained 51.9% of the variance based on the initial eigenvalues and 42.4% of the variance after the principal components were extracted. All six items were positively correlated with the identified factor, with factor loadings ranging from 0.608 to 0.685 and the extracted communality coefficients ranging from 0.370 to 0.469. Taken together with the reliability analysis, these results confirm the possibility of using the six questionnaire items as a single composite indicator of the perception of infrastructural capabilities (Table 14).

**Table 14:** Exploratory factor analysis of the pROI indicator based on six items.

Item/domain	Factor loading	Communality
Educational institutions	0.608	0.370
Medical institutions	0.685	0.469
Retail trade and public catering	0.646	0.418
Leisure and recreation	0.618	0.382
Sports facilities	0.683	0.466
Neighborhood safety	0.661	0.437

***Note:*** factorization by principal axes; one factor extracted. KMO = 0.852; Bartlett's χ^2^ (15) = 1317.358, *p* < 0.001. Initial eigenvalue = 3.116. The first factor explains 51.9% of the variance based on the initial eigenvalues and 42.4% after extraction.

#### 4.8.2 In terms of content, the deficit profile in a megalopolis has a stable hierarchy

Basic institutions are perceived to be the most accessible (complete “insecurity”: Education 8.2%, polyclinics 9.3%), while the maximum deficits are concentrated in the domains that shape the quality of daily life and routes – safety 20.3%, sports 18.9%, leisure 18.9%. This is an important result for Almaty: Barriers to opportunity arise not only where there is “no medicine,” but where the daily environment makes accessing services more costly and risky.

#### 4.8.3 The key “gradient of opportunity” runs along housing stability rather than formal neighborhood boundaries

Compared to living in your own apartment/house, living in a rented apartment is associated with a sharp increase in the proportion of “not provided at all” responses in the domains of the urban environment: Trade/food 24.1% vs 7.0%, sports/safety 25.1–26.1% vs 12.8–13.3% (rent vs property). At the association level, the type of accommodation shows small but stable links with infrastructure assessments (polyclinics *r* = 0.100; *p* = 0.002; sports *r* = 0.090; *p* = 0.006; safety *r* = 0.108; *p* = 0.001), whereas the area of residence is practically unrelated to infrastructure estimates (|r| ≈ 0.00–0.04; *p* > 0.05). In terms of the “Reproductive Opportunity Atlas,” this means: The pROI captures the micro-inequality of everyday conditions (micro-place stratification), which is more dependent on the trajectory of housing than on the administrative area as such.

Finally, the differences between regions in individual domains are uneven and are better seen by nonparametric tests: For a number of domains, statistical heterogeneity by region was revealed (Kruskal–Wallis: *p* = 0.025 for polyclinics; *p* < 0.001 for trade, leisure and sports; *p* = 0.013 for safety), while there were no differences in education (*p* = 0.844). This is consistent with the general logic of the index: In a megalopolis, the core of basic services is more aligned, and gaps are concentrated in the domains of urban daily life.

The pROI in Almaty reflects not just the infrastructure, but the structure of daily costs and secure opportunities, and the main fault runs through housing stability, which forms micro-contexts of access even within the same city.

## 5 Discussion

In this work, “place” is measured not through an inventory of objects, but through an experienced environment of possibilities. The pROI captures things that usually fall off the administrative charts: Secure routes, predictable access, hidden transaction costs (time, transport, waiting, the need to “pay extra” to get quality). This is fundamental for reproductive behavior: Decisions about visiting a doctor, choosing contraception, and postponing the birth of a child are often made not based on whether there is a service, but on how realistic and safe it is to receive it. Therefore, the perceptivity of the index here reflects not the noise, but the channel of the context.

### 5.1 Practical implications: pROI as a prioritization tool, not an index for the sake of an index

The key value of pROI is that it translates the “soft” layer of everyday experience into a management signal for planning. Unlike the inventory approach (“there is/is not an object”), pROI ranks territories according to the effective suitability of the urban environment for reproductive health: How realistic it is for a woman to get to help safely, on time and without excessive hidden costs (transportation, waiting, loss of working hours, repeated visits, the need to “pay extra” for quality). This is important for politics, because in a city with formally high coverage, the limiter is more often not the availability of services as such, but the conversion rate of coverage to real access. pROI allows you to see where this conversion is systematically “failing”.

This implies the direct application of the index to prioritize investments and sequence interventions. First, a low pROI indicates areas where the effect of “medical” investments will be limited if the basic shortage of daily safety is not addressed: lighting, safe routes, reduced risks of harassment and street anxiety, these are not peripheral improvements, but conditions for regular visits, commitment to monitoring and continued prevention. Secondly, pROI identifies gaps in the logic of proximity and reachability of services.: When “there is a polyclinic in the area” actually means disproportionately long travel time, fragmented transportation and time expenditure incompatible with real life schedules, especially for women in rental and unstable housing trajectories. Third, the index captures the lack of predictability (quality/time/cost): With formal availability of services, uncertainty and administrative complexity make it a rational strategy to avoid, postpone, or switch to one-time paid “bypass channels”. Therefore, pROI sets the logic not for disparate measures, but for targeted packages of security + access logistics + reliability of services, and thus serves as an operational tool for targeted planning of urban policies and services.

At the same time, the district as an administrative unit inevitably smooths out intra-district differences. The results should be read as a district-level diagnosis, rather than as a final spatial map: It is most likely that key gaps are located within districts (neighborhoods, rental clusters, transport corridors).

### 5.2 pROI as an index: consistency and semantic structure of deficits

Six aspects of the perceived urban environment demonstrated good internal consistency (Cronbach's alpha = 0.814; standardized Cronbach's alpha = 0.815; *n* = 780). The corrected correlations between the items and the total score ranged from 0.535 to 0.605, and the correlations between the items ranged from 0.327 to 0.517. Cronbach's alpha when individual items were removed ranged from 0.777 to 0.793, which indicates that removing any individual item did not increase the reliability of the measurement. These results confirm the internal consistency of the composite indicator consisting of six items.

Meaningfully, the picture of deficits looks not like there is no infrastructure at all, but like a hierarchy of the quality of urban daily life. The basic domains are rated better, and the maximum failures are due to what forms a daily vulnerability: Security (20.3% “not provided at all”), sports and leisure (18.9% each). This is an important clarification for a megalopolis: Reproductive opportunities may be limited not by the lack of clinics, but by the fact that the urban environment makes regular access to help costly and risky (Fig. 2).

**Fig. 2:**
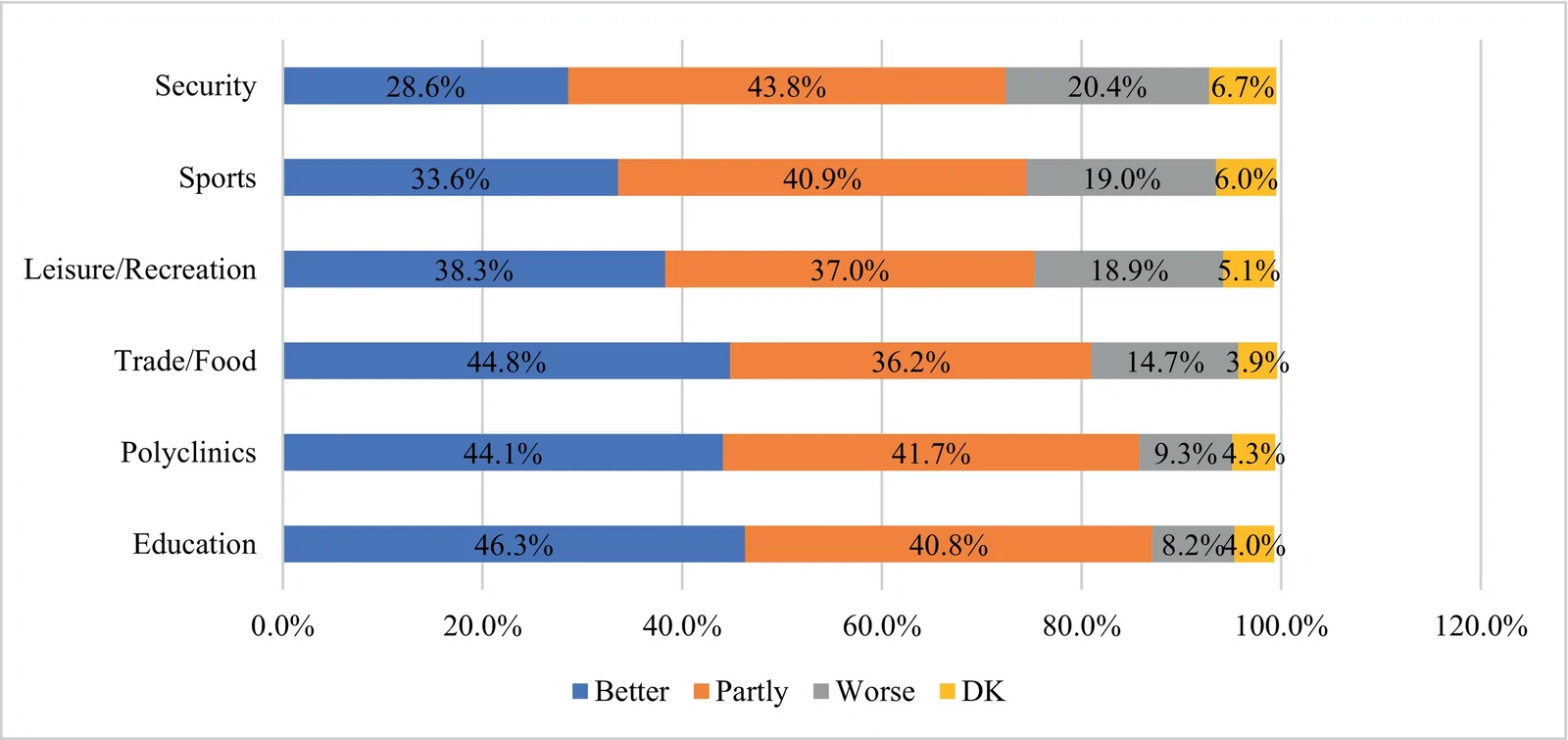
Assessment of changes in the quality of urban infrastructure in key areas, % (Better/Partly/Worse/DK).

The classical verification of regional averages (ANOVA) almost does not record stable differences, which is expected for ordinal scales and asymmetric distributions. However, nonparametric comparisons show that districts differ in distribution/rank: Significant differences were found for polyclinics (*p* = 0.025), retail and food services (*p* < 0.001), leisure (*p* < 0.001), sports (*p* < 0.001) and safety (*p* = 0.013), while there were no differences in education. (*p* = 0.844). After correcting for multiple comparisons, the key conclusion remains: Geography manifests itself primarily as an everyday environment, and not in the basic core of services.

However, the main fault that makes these differences socially significant is not related to the area in general, but to the housing trajectories in which women live. Almaty demonstrates a typical configuration for a megalopolis: The “place” works through housing stability, not just through administrative boundaries. This is micro-place stratification: People can formally live in the same area, but actually in different access modes.

The central empirical result of the article is the resource-based nature of access barriers. In the multidimensional model of financial constraints on access to reproductive health services, the overall contribution of the model is significant (LR χ2(136) = 253,56; *p* < 0,001; Nagelkerke R^2^ = 0,251). The most stable factor remains the household's material well-being (LRT for the welfare effect: *p* < 0.001), and this pattern is reproduced in several outcome categories. This is consistent with bivariate estimates: The association of well-being with financial failures is statistically pronounced (χ2 = 101.19; *p* < 0.001; V = 0.165), whereas the district factor is weaker (χ2 = 44.94; *p* = 0.022; V = 0.110) and more sensitive to multiple comparisons.

The meaning here is more important than the wording poverty affects: In an urban context, where formal coverage of services may look high, inequality is reproduced through the ability to pay for the invisible cost of access to paid examinations, medicines, transport, time, alternative quality channels. It is especially significant that the do not apply strategy is more common among the less well-off: This may not reflect a lack of need, but rather an accumulated experience of unavailability, distrust, and expectations of high costs.

Education also makes a significant contribution (*p* < 0.001), which indicates that barriers are structured not only by resources, but also by human capital (navigation through the system, knowledge of routes, the ability to convert information into action). At the same time, the interpretation of individual coefficients for rare categories requires caution due to sparsity and warnings about the instability of estimates; therefore, it is more correct to focus on stable contributions of factors (LRT) rather than on the exact size of effects for rare combinations.

Well-being is associated with the structure of current contraceptive practices (χ2 = 143.81; *p* < 0.001; V = 0.176) and less with the postponement of childbirth (χ2 = 28.23; *p* = 0.020; V = 0.101). It is more correct to interpret these results as a differentiation of available strategies, rather than as a juxtaposition of responsible and irresponsible ones. With limited resources, the cost of mistakes and the proportion of decisions dictated by forced savings and uncertainty are increasing. Therefore, in a megalopolis, reproductive behavior becomes not just a choice, but risk management in an environment where access costs are unevenly distributed.

The main contribution of the work is the empirical fixation of the fact that in Almaty, a place acts as a mechanism of hidden costs, and not as a simple map of districts: The household resource is stronger than pure geography, and place effects are more subtle through the quality of the daily environment and housing stability. This clarifies the international discussion about the paradox of coverage: Even with the apparent availability of services, there are persistent gaps in chances and practices, because the availability of quality and predictability is unevenly distributed.

Generalization is possible for other large cities of Kazakhstan and comparable post-Soviet megacities with a high proportion of rentals and intra-urban segregation, however, the study design is cross-sectional, the data is self-reporting, and some categories are sparse. Therefore, the conclusions are formulated as associative; the next step is more subtle spatial validation (neighborhoods, transport accessibility, administrative data, geocoding).

## 6 Limitations

The present study has a number of limitations typical of works on socio-economic determinants of reproductive behavior, and their consideration is important for the correct interpretation of the results.

Firstly, the design of the study is cross-sectional: It reliably captures associations and social gradients, but does not allow making causal conclusions and tracking changes over time.

Secondly, the indicators are based on self-reports from female respondents, so the risks of socially desirable responses and recall errors remain, especially for sensitive topics.

Thirdly, the empirical base reflects the conditions of the city of Almaty; therefore, the transfer of conclusions to other regions requires caution due to differences in infrastructure, accessibility of services and socio-cultural contexts.

Fourth, the financial situation of a household is measured by a well-being survey indicator, which reflects well the standard of living of resources, but is not fully equivalent to administratively measured income.

Finally, although the study uses the logic of microstratification of opportunities (in the spirit of Opportunity Atlas), it does not build a full-fledged causal model of territorial effects and does not rely on long-term administrative trajectories, this remains the prospect of further research.

An additional limitation is the spatial aggregation of data: Analysis at the district level inevitably smooths out intra-district differences between neighborhoods and local “access routes” (security, travel time, real accessibility of services). Therefore, inter-district effects should be interpreted as a characteristic of the overall macro context of opportunities, rather than as an accurate diagnosis of specific points of scarcity. A promising area of development is to increase spatial accuracy, the transition to micro-level units and geocoding (place of residence / path to services) with the addition of accessibility indicators and, if possible, in conjunction with data on the actual use of services; this will enhance the applied targeting of conclusions.

At the same time, the limitation in detail does not make the work “only theoretical”: The proposed framework for the geography of reproductive opportunities and the pROI index already function as a practical tool for primary targeting. The pROI allows you to rank territories by perceived infrastructural support and identify areas where priority is given to measures for the safety of everyday routes, accessibility and predictability of services, and the development of local infrastructure. In other words, the current model sets a clear logic for “where to direct efforts first,” and increasing spatial accuracy will make this tool even more accurate for urban policy and service planning.

## 7 Conclusion

This article began with a question: How can reproductive inequality persist where formal coverage looks almost “ideal”? Kazakhstan is indeed approaching its maximum in terms of coverage of obstetric care and maternity services, but the shock volatility of maternal mortality in 2020-2021 and persistent territorial gaps show that the problem lies not in the availability of services, but in the distribution of timeliness, quality and cost of access. In this sense, Almaty is not an exception, but a laboratory: The metropolis concentrates infrastructure, specialists and the private sector, but at the same time produces more subtle and elusive forms of exclusion from the system.

The main empirical conclusion of the study can be stated in plain text: In Almaty, women's reproductive opportunities are structured by a double contour, that is, household resources and the microgeography of everyday life. At the first level, material well-being is crucial: It is statistically consistently related to the financial barriers to accessing reproductive health services, the configuration of contraceptive practices, and (albeit weaker) the reasons for postponing the birth of a child. This is not a truism about “poverty”, but about the fact that access in a megalopolis turns into a cost market: Money is needed not only for admission, but also for time, transportation, examinations, medicines, repeat visits, quick login and reduced uncertainty. Therefore, the socio-economic position of a household in a city works as a functional equivalent of infrastructure: It determines whether a woman will be included in the preventive contour or will find herself in a mode of forced postponement and self-restraint.

At the second level, the study reveals something that is often not seen in administrative geography: The place-effect in a megalopolis manifests itself not so much through neighborhoods as through the housing trajectory. The “property vs rent” divide turned out to be more informative for understanding the perceived possibilities of the urban environment than the formal boundaries of the districts. This is important for the international reader: We show a typical mechanism of modern big cities, but based on the material of a post-Soviet megacity, where the housing market and infrastructure are developing faster than social protection systems and urban justice. In such a city, you can live in the same area, but in different reality modes: The same administrative contour includes secure courtyards and vulnerable rental clusters, where the daily price of mobility is higher, and the choice is always made under pressure.

This is where the meaning of the pROI index becomes clear. The Perceived Reproductive Opportunity Index in this work is not an attempt to replace “objective” maps with subjective opinions and is not a demonstration of methodological skill. This is an attempt to capture something that rarely gets into statistics, but affects decisions on a daily basis: Route safety, predictability of access, the ability to reach a service without additional anxiety and losses, that is, hidden transaction costs that especially affect women. The resulting hierarchy of deficits is important in itself: The basic institutions (education, primary care) are perceived more sustainably, and the maximum failures are concentrated in the domains of quality of daily safety, sports and leisure. This suggests that in a megalopolis, reproductive autonomy is limited not only by medicine as such, but also by the viability of the urban environment: If it is impossible to safely move and recover, then any prevention becomes more expensive and less regular.

The contribution of the article is not that we “confirmed that income is important.” The contribution is different: We show exactly how inequality is reproduced in a city with formally high security. In Almaty, there are differences between districts in terms of financial constraints, but they are relatively small and partially disappear when taking into account the composition of the population. This does not mean that a place is not important; it means that in a megalopolis, a place acts more subtly through microstratification and housing stability, rather than through rough administrative boundaries. Thus, the article adds a key clarification to the international discussion about the paradox of coverage: Universality of coverage can coexist with non-universality of opportunities, because opportunity is not the fact that a service is available, but the ability to use it stably and safely.

This leads to applied conclusions that make the work alive and politically significant. If inequality manifests itself as different modes of inclusion in services from prevention to the no appeal strategy, then policy should not be limited to the slogan “increase coverage”. We need measures that reduce the hidden cost of access, especially for women in vulnerable housing and resource trajectories. Practically, this means:

− Targeting not only by districts, but also by clusters of housing precarity (rental zones, dormitories, unstable accommodation formats);
− Reduction of out-of-pocket costs precisely at critical points of the route (consultation → examinations → medications → repeat visit), otherwise the free system remains partially paid in practice;
− Strengthening urban daily life as conditions for reproductive health: Lighting, safety, transport accessibility, convenient routes to clinics and family planning centers, this is not urbanism “aside from medicine”, but part of reproductive justice.

Finally, the article formulates the perspective of the development of the Reproductive Opportunity Atlas as the next stage of research and practice. In the current version, we have taken an operational step by showing that the even survey-based proxy can capture micro-inequalities of opportunity and link them to reproductive practices. The next level is to combine this logic with more subtle data: neighborhoods, geocoding, transport accessibility, administrative service quality indicators, as well as repeated measurements that allow you to separate the effect of a place from the effect of life events. This does not negate the value of the current results; on the contrary, they provide a map of hypotheses, where to look for hot spots of inequality and which mechanisms to check next.

As a result, the meaning of the work can be expressed in one phrase: In a megalopolis, reproductive decisions are not only an intimate choice and not only a medical outcome, but a form of risk management in conditions of unequal distribution of opportunities. Almaty shows that even with high formal accessibility, the system can remain selective in terms of real access, through money, time, housing and the quality of the daily environment. And if we want to talk about reproductive justice seriously, we must measure not only coverage, but also opportunities: Who gets a chance at safe family and maternity planning, where, and at what cost.

## AI disclosure

During the manuscript preparation process, the authors used a generative artificial intelligence tool to a limited extent for editorial assistance, including improving the clarity, structure, and style of individual text fragments. The authors generated the research idea, developed the methodology, conducted the analysis, interpreted the results, and formulated the scientific conclusions independently. The authors fully reviewed and revised the text after using this tool and bear full responsibility for the content of the article.

## CRediT authorship contribution statement

**Ainur Bakytzhanova:** Writing – review & editing, Writing – original draft, Visualization, Validation, Supervision, Software, Resources, Project administration, Methodology, Investigation, Formal analysis, Data curation, Conceptualization. **Gulzhan Alimbekova:** Validation, Data curation.

## Informed consent

All participants provided voluntary informed consent to participate in the study. Data collection was conducted under the conditions of anonymity and confidentiality; participants could withdraw from participation at any stage without any consequences.

## Ethical approval

The study was conducted in accordance with international and professional ethical standards, including the Declaration of Helsinki, the Oviedo Convention, the UNESCO Declaration on Bioethics and Human Rights, and the ISA, RSS, and ICC/ESOMAR ethical codes. The project was approved by the Local Ethics Committee of Al-Farabi Kazakh National University (No. IRB–A1594 dated April 17, 2025).

## Funding

This study did not receive any specific grants from funding agencies in the public, commercial, or not-for-profit sectors.

## Declaration of competing interest

No conflicts of interest were identified. The authors declare no financial, professional, or other circumstances that could affect the objectivity of the presented study.

## Data Availability

The data cannot be made publicly available due to ethical restrictions and the need to protect the privacy of participants. Anonymized aggregated results are available in the article.
